# MH002, a Novel Butyrate-Producing Consortium of Six Commensal Bacterial Strains Has Immune-Modulatory and Mucosal-Healing Properties

**DOI:** 10.3390/ijms26136167

**Published:** 2025-06-26

**Authors:** Iris Pinheiro, Selin Bolca, Lien Van den Bossche, Wiebe Vanhove, Sara Van Ryckeghem, Davide Gottardi, Debby Laukens, Sam Possemiers

**Affiliations:** 1 MRM Health NV, Technologiepark 82, 9052 Ghent, Belgium; selin.bolca@gmail.com (S.B.); wiebe.vanhove@imec.be (W.V.); davide.gottardi2@unibo.it (D.G.);; 2 Department of Chronic Diseases, Metabolism & Ageing (CHROMETA), Translational Research Center for Gastrointestinal Disorders (TARGID), KU Leuven, 3000 Leuven, Belgium; 3 IBD Research Unit, Ghent Gut Inflammation Group (GGIG), Department of Internal Medicine and Pediatrics, Ghent University, 9000 Ghent, Belgium

**Keywords:** IBD, ulcerative colitis, bacterial consortium, butyrate, intestinal barrier, anti-inflammatory, mucosal repair

## Abstract

Inflammatory bowel disease (IBD) is a chronic relapsing inflammatory condition of the gastrointestinal tract. It is generally accepted that IBD is characterized by an inappropriate immune response to the intestinal microbiome in genetically susceptible individuals. Despite the available treatment options ranging from salicylates and corticosteroids, to immunosuppressants and biologics, there is still a high unmet medical need for patients who respond poorly to drugs or are not able to tolerate them. Microbiome-based therapeutics offer a valid treatment strategy for IBD with enhanced safety. A butyrate-producing consortium of six commensal strains (MH002) was evaluated in a series of in vitro, ex vivo, and in vivo experiments mimicking multiple IBD-related dysfunctions, namely disrupted intestinal permeability and immune activation. MH002 rapidly produced high levels of butyrate in fed-batch cultures, and significantly increased butyrate levels within one day after administration to IBD-derived gut microbial communities in vitro. Both in Caco-2/peripheral blood mononuclear cells (PBMCs) co-cultures, and IBD patients-derived organoids and colonic explants, MH002 reduced inflammation and restored epithelial barrier integrity. In addition, MH002 promoted wound repair in vitro. Finally, MH002 protected mice and rats from chemically induced colitis. Altogether, results showed that MH002 presents a novel therapeutic avenue for the treatment of IBD.

## 1. Introduction

Inflammatory bowel disease (IBD) is a chronic inflammatory disease of the gastrointestinal (GI) tract and consists of two major subtypes: Crohn’s disease (CD) and ulcerative colitis (UC). In CD, inflammation can occur anywhere along the GI tract, whereas in UC, disease is limited to the large intestine and rectum. Although considered idiopathic, IBD is a multifactorial disease influenced by genetic and epigenetic factors, environment, gut microbiota, and immune system [1]. IBD is a contemporary disease of industrialized societies, associated with westernization of diets and environment, which in turn influence the gut microbiome composition and increase the risk for disease in genetically susceptible individuals [2,3]. In mild-to-moderate UC, the first-line treatment is 5-Aminosalicylic acid (5-ASA), which may be used to induce remission as monotherapy or in combination with corticosteroids or immunomodulators [4]. However, a proportion of patients do not respond to 5-ASA or fail to remain in remission, thereby requiring step-up therapy to other agents such as biologics that are immunosuppressive by nature, and therefore associated with unwanted side effects, such as increased risk for infections [5]. Overall, nearly 50% of patients require hospitalization and 10–15% require colectomy after 10 years [6]. Additional treatment options that can either delay the use of immunosuppressive agents or improve clinical efficacy remain of high interest [7,8].

The gut microbiome of IBD patients is significantly different from that of healthy subjects [9]. Such alteration of the gut microbial environment is commonly referred to as ‘dysbiosis’, and this is involved in the pathophysiology of IBD [10]. The notion that gut microbial dysbiosis is at the onset of IBD is even further strengthened by the fact that germ-free mice, upon inoculation with IBD-derived microbiota, develop colitis [11,12]. The gut microbiome has numerous functions, among which are nutrition, immune modulation, metabolism, and defense against pathogens. Short-chain fatty acids (SCFAs), including acetate, propionate, and butyrate, are the main bacterial metabolites produced upon carbohydrate fermentation, and are of major importance for the maintenance of gut immune and metabolic homeostasis [13]. Butyrate, an important energy source for colonocytes, is consistently reduced in the gut of IBD patients with active disease [14]. Butyrate not only supports colonic function but has also numerous proven effects that provide health benefits [15]. Among others, butyrate has anti-inflammatory effects and promotes intestinal barrier function and mucosal immunity [16]. Levels of butyrate and substrates involved in butyrate synthesis have been described to be significantly associated with clinical remission following anti-tumor necrosis factor (TNF) therapy [17]. In IBD patients, the levels of butyrate producers such as *Eubacterium rectale*, *Faecalibacterium prausnitzii*, and *Roseburia intestinalis* are reduced, whereas the relative abundance of pathogens such as adherent-invasive *Escherichia coli*, and opportunistic bacteria such *as Ruminococcus gnavus*, are increased [18,19,20,21]. Because gut microbial dysbiosis is an important pathophysiological feature in IBD, modulation of the gut microbiome holds promise and may complement the currently available therapeutic armamentarium [22]. In support of this, positive effects of fecal microbiota transplantation (FMT) on disease activity have been reported [23], yet with variable efficacy, which is likely linked to the inherent variable composition of FMT and complex delivery strategy. Therefore, live biotherapeutic products (LBPs), which are based on isolated and well-characterized bacterial strains, provide an alternative to FMT for the treatment of gut inflammatory diseases. Defined combinations of bacteria (consortia) are typically well-characterized and safe and can be manufactured under standardized pharmaceutical conditions. In line with this, we developed MH002, an LBP consisting of six well-characterized bacterial strains, all members of the core human gut microbiome and/or associated with mucosal protection and immune-modulatory effects.

In this study, we have assessed the capacity of MH002 to increase butyrate levels in fed-batch cultures and in the Simulator of the Human Intestinal Microbial Ecosystem (SHIME^®^) upon inoculation with IBD-derived dysbiotic microbial communities. We have also tested cell-free supernatants collected from these fermentations in both Caco-2 cells and intestinal organoids derived from IBD patients. The effect on the release of immune mediators was investigated in human peripheral blood mononuclear cells (PBMCs) and patient colonic explants. Finally, rodent studies were conducted to confirm the therapeutic potential of MH002 to improve colitis. Altogether, results demonstrated that MH002 is a valid therapeutic option for IBD patients.

## 2. Results

### 2.1. SCFA Production in the Presence and Absence of IBD Fecal Microbiome

**Figure 1:**
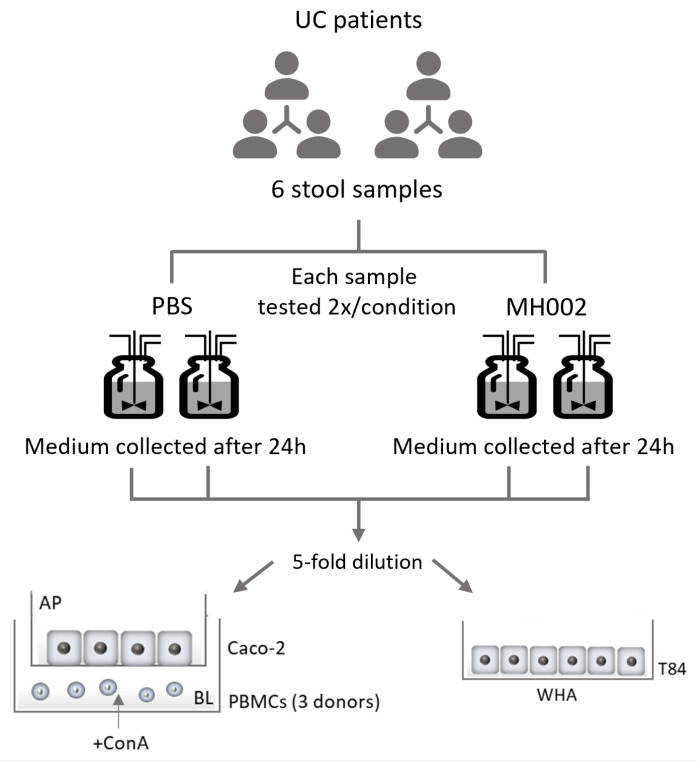
Experimental layout of the Simulator of the Human Intestinal Microbial Ecosystem (SHIME^®^) experiment (IBD-M-SHIME). Stool samples from six ulcerative colitis (UC) patients were added to the SHIME reactors in a 2 × 2 configuration, i.e., each stool sample was ‘treated’ with either MH002 or phosphate-buffered saline (PBS) in duplicate. Then, each reactor was sampled on day one, and the collected cell-free supernatants were tested in cell culture assays. For Caco-2/peripheral blood mononuclear cells (PBMCs) co-cultures, this was repeated in three independent experiments, with PBMCs isolated from three different (healthy) subjects and seeded on the basolateral (BL) side of semi-permeable inserts. The collected cell-free supernatants were 5-fold diluted (*v*/*v*) in a cell culture complete medium and added apically (AP) to the Caco-2 cells for a total of 48 h. Each reactor sample was tested in duplicate. All conditions were normalized to the average of the stimulated concanavalin A (ConA) control per experiment (i.e., per PBMC donor). For the wound healing assay (WHA) in T84 cells, cell-free supernatants collected from both PBS- and MH002-inoculated reactors were 5-fold diluted (*v*/*v*) in serum-free cell culture medium and added to the cells for 48 h. Each reactor sample was tested in triplicate. The experiment included six plates (one plate per stool sample), and each plate contained three control wells treated with serum-free medium (untreated cells control). To account for inter-plate variation, wound closure of treated cells was normalized to the average of medium (untreated cells) control per plate.

To assess the fermentation capacity of MH002 and to obtain samples for in vitro cell cultures, MH002 was tested in two different systems: in fed-batch cultures containing only nutritional medium, or in the Simulator of the Human Intestinal Microbial Ecosystem (SHIME^®^) inoculated with fecal samples obtained from six ulcerative colitis (UC) patients (Figure 1).

**Figure 2:**
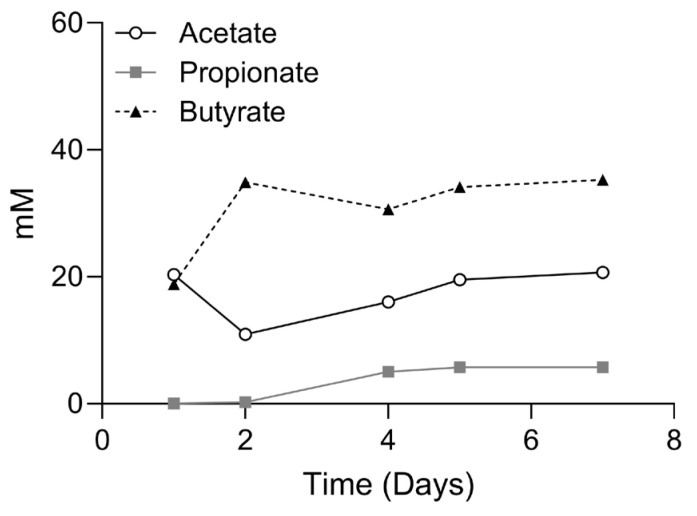
MH002 fermentation capacity in fed-batch cultures. Lyophilized MH002 was added to one bioreactor containing nutritional medium. MH002 was kept in culture for seven days under anaerobic and pH-controlled conditions. Samples were collected at regular intervals to measure short-chain fatty acids (i.e., acetate, propionate, and butyrate), and at day four for evaluation in in vitro cell cultures.

Upon inoculation of freeze-dried MH002 to fed-batch cultures, the levels of butyrate rapidly increased: after two days these were >30 mM and remained so until the end of the experiment (day 7). In contrast, acetate levels decreased after two days, indicating efficient conversion of acetate into butyrate by MH002 strains in fed-batch fermentations (Figure 2). On day four, samples were collected for in vitro cell cultures. The concentrations of short-chain fatty acids (SCFAs) present in the tested sample were 16.0, 5.0, and 30.6 mM of acetate, propionate, and butyrate, respectively. Upon 5- or 7-fold dilution in cell culture medium, the levels varied between 2.3 and 3.2, 0.7 and 1.0, and 4.4 and 6.1 mM of acetate, propionate, and butyrate, respectively.

**Figure 3:**
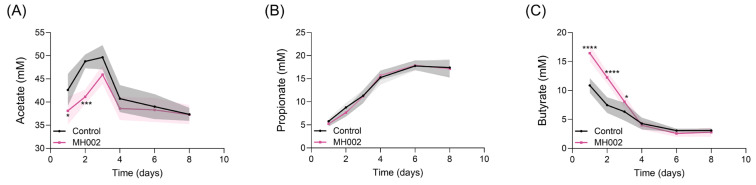
Effect of MH002 or vehicle control phosphate-buffered saline (PBS) on short-chain fatty acid production under in vitro conditions simulating the colonic environment of ulcerative colitis patients in the IBD-M-SHIME^®^. (**A**) Acetate; (**B**) propionate; and (**C**) butyrate levels measured throughout the experiment (*n* = 6 per condition). Samples were collected on day one for in vitro cell cultures. Data are presented as the mean ± SE of the mean of six (stool) UC samples (shaded areas). In all plots, asterisks indicate significant difference *, *p* < 0.05; ***, *p* < 0.001; and ****, *p* < 0.0001 between control and MH002 within each sampling point (two-way repeated measures ANOVA with Šídák’s post hoc test).

In the presence of stool microbiome collected from six UC patients, a single dose of MH002 led to the production of significantly higher butyrate levels after 24 h (16.45 ± 1.42 mM, MH002 vs. 10.87 ± 1.28 mM, PBS control, *p* < 0.0001), in the UC-like simulated colonic environment and remained significantly higher when compared to the PBS-treated SHIME^®^ for the first three days, whereas acetate levels remained lower. After that, and from day four onwards, both butyrate and acetate levels decreased (Figure 3A,C). Propionate levels increased throughout the experiment but were not different between PBS- and MH002-treated conditions (Figure 3B). Samples collected on day one were used for in vitro cell cultures. Upon 5-fold dilution in cell culture medium, the SCFA levels for the control SHIME^®^ were on average 8.5, 1.2, and 2.2 mM of acetate, propionate, and butyrate, respectively, whereas for MH002-treated reactors, these were 7.6, 1.1, and 3.3 mM of acetate, propionate, and butyrate, respectively.

### 2.2. Effect of MH002 on Intestinal Barrier Integrity and Cytokine Release In Vitro

**Figure 4:**
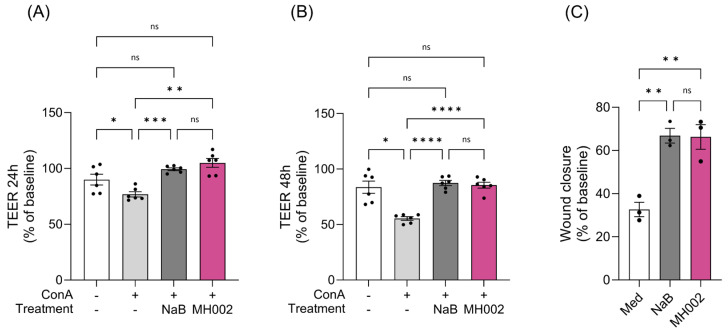
Effect of MH002 cell-free supernatant and 5 mM sodium butyrate (NaB) on intestinal barrier integrity of Caco-2/PBMCs co-cultures and wound closure in T84 cells. Transepithelial electrical resistance (TEER) of Caco-2/PBMCs co-cultures after (**A**) 24 and (**B**) 48 h of treatment (*n* = 6 PBMC donors per condition; these correspond to the black dots). Data are presented as the mean ± SE of the mean of PBMCs isolated from six different subjects and tested in three independent experiments. (**C**) Wound closure in T84 cells after 24 h (*n* = 3 replicates). Data are presented as mean ± SE of the mean of three experimental replicates. In (**A**,**B**), asterisks indicate significant difference *, *p* < 0.05; **, *p* < 0.01; ***, *p* < 0.001; and ****, *p* < 0.0001 after one-way repeated measures ANOVA with Tukey’s post hoc test, and ns indicates not significant. In (**C**), asterisks indicate significant difference **, *p* < 0.01 after (ordinary) one-way ANOVA with Tukey’s post hoc test, and ns indicates not significant.

**Figure 5:**
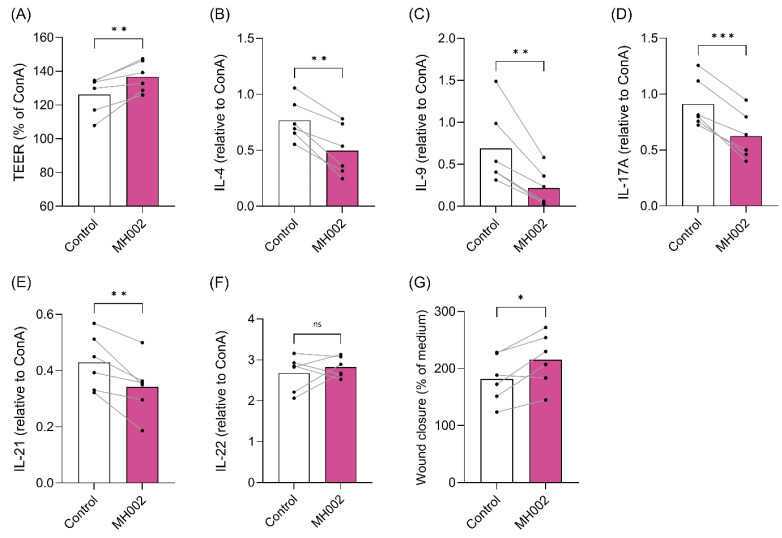
Effect of MH002 or vehicle control (PBS) on intestinal barrier integrity, cytokine release, and wound repair under in vitro conditions simulating the colonic environment of six UC patients in the IBD-M-SHIME^®^ (**A**) TEER of Caco-2/PBMCs co-cultures after 48 h (*n* = 6 stool UC donors per condition; these correspond to the black dots). (**B**–**F**) Cytokine levels measured on the basolateral compartment of Caco-2/PBMCs co-cultures (*n* = 6). Results are shown as paired data (per patient-stool) and are presented relatively to the average of ConA and the mean of six (stool) UC samples. (**G**) Wound closure in T84 cells measured after 48 h. Results are shown relative to untreated cells and present the mean of six (stool) UC samples (paired data). In all plots, asterisks indicate significant difference *, *p* < 0.05; **, *p* < 0.01; ***, *p* < 0.001 after paired Student’s *t*-test, and ns indicates not significant.

To assess the effects of MH002 on intestinal barrier integrity and cytokine release, MH002 cell-free supernatants collected from either the fed-batch cultures (Figure 4 and Table 1) or from the IBD-M-SHIME^®^ (Figure 5) were administered to Caco-2/peripheral blood mononuclear cells (PBMCs) co-cultures. In both experiments, sodium butyrate (NaB) was tested alongside for comparison purposes. As expected [24,25], a reduction in transepithelial electrical resistance (TEER), a measure of paracellular permeability, was observed in Caco-2 cells upon co-culture with activated immune cells: a reduction of approx. 23% and 45% was observed upon co-culture with concanavalin A (ConA)-stimulated PBMCs, after 24 and 48 h, respectively (Figure 4A,B). Upon apical treatment with MH002 cell-free supernatant, the TEER significantly increased to similar levels as observed for medium-treated co-cultures, i.e., to 105% (*p* = 0.002) and 86% (*p* < 0.0001) after 24 and 48 h, respectively (Figure 4A,B). This indicated that MH002 was able to rescue Caco-2 barrier integrity from the decrease induced by activated PBMCs. The effects were comparable to 5 mM sodium butyrate.

The stimulation of PBMCs in the basolateral chamber induces an increase in cytokine release, which is dependent on the stimulus used [26]. Here, we have also observed a significant increase in cytokines released upon stimulation with ConA (Table 1). ConA is a T cell mitogen, which induces the release of T cell prototypical cytokines [26]. Upon treatment with MH002 cell-free supernatant, a significant reduction was observed for interleukin (IL)-4 (*p* = 0.04), IL-17 (*p* = 0.01), and IL-21 (*p* = 0.008). For IL-9, despite the observed reduction, it did not reach statistical significance. In contrast, IL-22 levels increased in MH002-treated cells when compared to ConA. The inhibitory or stimulatory effects induced by MH002 were similar to those observed for 5 mM sodium butyrate, or often more pronounced (e.g., IL-4, IL-21). To assess whether the different treatments could reduce cell viability, the activity of lactate dehydrogenase (LDH) was measured on the basolateral compartment. No statistically significant differences were observed when compared to ConA, thus indicating that reduction in cytokine levels could not be attributed to a decrease in cell viability (Table 1).

A similar effect was observed upon treatment with IBD-M-SHIME^®^-collected samples: although an increase in TEER was observed for control (PBS)-treated SHIME^®^ samples (26%), the luminal samples collected after inoculation with MH002 induced a more pronounced increase in TEER (37%) in Caco-2/PBMCs co-cultures, which was significantly different when compared to control-treated SHIME^®^ samples (*p* = 0.01, Figure 5A).

For comparison purposes, 2.5 and 5 mM sodium butyrate were tested alongside the same cellular experiments (Figure S1). The protective effects were found to be dose-dependent: despite lack of significance, 2.5 mM sodium butyrate induced an increase in the TEER of approx. 6%, and 5 mM induced an increase of 35% (Figure S1A), which is comparable to the results obtained for MH002. In addition, while basolateral levels of IL-4, IL-9, IL-17A, IL-21, and IL-22 increased upon ConA stimulation of the cells (Table S1), IL-4, IL-9, IL-17, and IL-21 significantly decreased upon treatment of the cells with the luminal samples from the IBD-M-SHIME^®^ inoculated with MH002, when compared to donor-matched vehicle control samples (*p* = 0.001, *p* = 0.006, *p* = 0.0003, and *p* = 0.01 for IL-4, IL-9, IL-17, and IL-21, respectively; Figure 5B–E). In contrast, IL-22 levels showed a variable response across the different IBD stool donors and were not significantly different between MH002 and PBS control (Figure 5F). Effects were also comparable to those of cells treated with 5 mM sodium butyrate (Figure S1B–F).

### 2.3. Effect of MH002 on In Vitro Wound Repair

MH002 cell-free supernatants were tested in an in vitro ‘scratch’ assay. This was performed in T84 cells, which have a colonocyte-like phenotype [27]. In this assay, cells naturally migrate to close the mechanically inflicted wound [28]. Also in the current study, medium-treated cells were able to close the ‘scratch’ to some extent (~30% after 24 h), but MH002 had a clear enhancing effect: after 24 h the ‘scratch’ was closed for about 66%, an effect significantly higher when compared to medium-treated cells (*p* = 0.004, Figure 4C). A similar effect was observed for cells treated with the samples collected from the IBD-M-SHIME^®^: luminal samples collected after inoculation with MH002 significantly enhanced wound closure in T84 colonocytes when compared to the PBS control (18% increase, *p* = 0.02, Figure 5G). In both experiments, results were comparable to 5 mM sodium butyrate (Figure 4C and Figure S1G).

### 2.4. Effect of MH002 on Biopsy-Derived Intestinal Organoids and Colonic Explants

**Figure 6:**
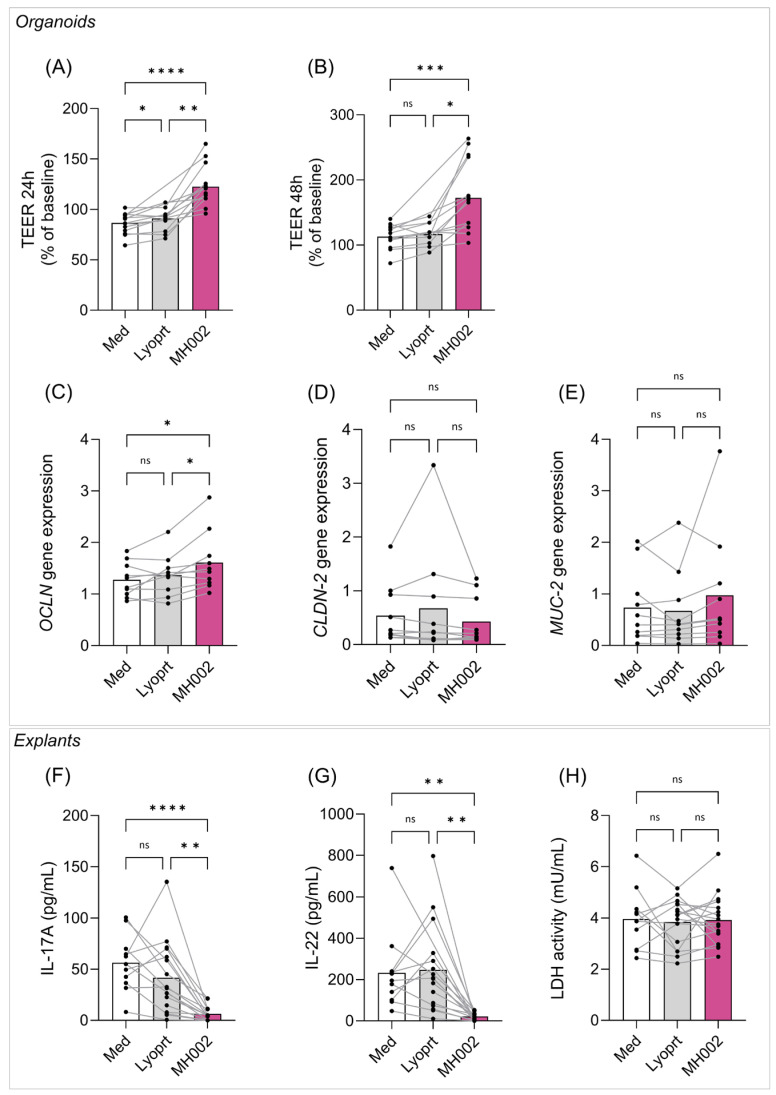
Effect of MH002 cell-free supernatant on IBD patients-derived (**A**–**E**) intestinal organoids and (**F**–**H**) colonic explants when compared to lyoprotectant (Lyoprt). The TEER was measured after (**A**) 24 and (**B**) 48 h of treatment (*n* = 14 patient organoids for Med and MH002; *n* = 12 for Lyoprt). (**C**–**E**) Expression of tight junctions’ genes at the end of treatment (*n* = 10 patient organoids for all groups). Data are shown as paired data (per patient’s biopsy) and are presented as the mean of 10–14 IBD patients. (**F**,**G**) Cytokine levels and (**H**) LDH activity were measured in colonic explants supernatant after anti-CD3 treatment (*n* = 11, *n* = 16, and *n* = 17 explants for Med, Lyoprt, and MH002, respectively). Data are shown as paired data (per patient-explant) and are presented as the mean of 11–17 IBD patients. Asterisks indicate significant difference *, *p* < 0.05; **, *p* < 0.01; ***, *p* < 0.001; and ****, *p* < 0.0001 after one-way repeated measures ANOVA or matched mixed-effects analysis and Tukey’s post hoc test, and ns indicates not significant.

To further validate the observed effects, experiments were performed using biopsies collected from IBD patients ex vivo. For that, MH002 cell-free supernatant was tested in biopsies collected from IBD patients (Figure 6). Briefly, colonic mucosal biopsies were collected from IBD patients with active disease. In total, 21 patients were included in this study (11 UC, 10 CD; 10 males, 11 females), with a median age of 42 years old. Biopsies were collected for either intestinal crypts’ isolation or used as colonic explants. Organoids were successfully derived from the crypts of 14 patient biopsies, and colonic explants from 17 patient biopsies (11 patients were common to both experiments). For these experiments, freeze-dried MH002 was grown in fed-batch cultures and samples were collected after four days. This sample contained 14.7, 5.6, and 35.5 mM of acetate, propionate, and butyrate, respectively. These levels were comparable to the ones measured in the fed batch prepared for in vitro cell cultures (see above). Upon 7-fold dilution in cell-culture medium levels were 2.1, 0.8, and 5.1 mM of acetate, propionate, and butyrate, respectively.

#### 2.4.1. Effect of MH002 on IBD Patients-Derived Organoids

Organoids contain colonocytes and, among others, goblet cells expressing mucin (MUC)-2. When placed on semi-permeable membranes, a polarized monolayer is formed, allowing the study of barrier function and integrity [29]. The polarized monolayers were treated with either cell culture medium (Med), MH002-, or lyoprotectant-cell-free supernatants. The integrity of the intestinal barrier on the organoids was evaluated by measuring the TEER (Figure 6A,B). After 24 h, MH002-treated monolayers showed a significant increase in TEER, when compared to medium- and lyoprotectant-treated cells [42% (*p* < 0.0001) and 35% (*p* < 0.001) increase, respectively], which further increased after 48 h [53% (*p* = 0.0005) and 47% (*p* = 0.012) increase, when compared to medium and lyoprotectant, respectively]. To further characterize this protective effect on barrier integrity at the molecular level, organoids were collected after 48 h of treatment, and expression levels of occludin (*OCLN*), claudin (*CLDN*)*-2*, and *MUC-2* were measured (Figure 6C–E). Expression of *OCLN*, described to promote barrier tightening, showed a significant increase when compared to both medium and lyoprotectant (*p* = 0.018 and *p* = 0.029, respectively; Figure 6C). While not reaching significance, the expression of *MUC-2* increased in MH002-treated organoids (Figure 6E), and *CLDN-2* expression, a pore-forming claudin described to be upregulated in IBD and contributing to diarrhea [30], decreased (Figure 6D).

#### 2.4.2. Effect of MH002 on IBD Patients-Derived Colonic Explants

In contrast to organoid preparations, colonic explants contain immune cells and when combined with specific stimuli, they can be used to study immunological responses ex vivo [31]. Therefore, to evaluate the immunological response to MH002 cell-free supernatant, IBD patients-derived colonic explants were co-treated with samples collected from the fed-batch cultures and anti-CD3. No cytokines were detected when the explants were treated with the samples in the absence of anti-CD3. After 24 h co-treatment with anti-CD3, only IL-17 and IL-22 were reliably measured. IL-4, IL-9, IL-12p70, IL-21, and interferon (IFN)γ were below the level of detection. Upon anti-CD3 exposure, both IL-17 and IL-22 levels increased in the medium and lyoprotectant conditions (Figure 6F,G), yet they were significantly reduced upon MH002 treatment, without affecting cell viability (Figure 6H) (IL-17, *p* = 0.0001 and *p* = 0.002 when compared to medium and lyoprotectant, respectively; IL-22, *p* = 0.01 and *p* = 0.002 when compared to medium and lyoprotectant, respectively).

### 2.5. Effect of MH002 in Chemical-Induced Acute Colitis in Rats and Mice

**Figure 7:**
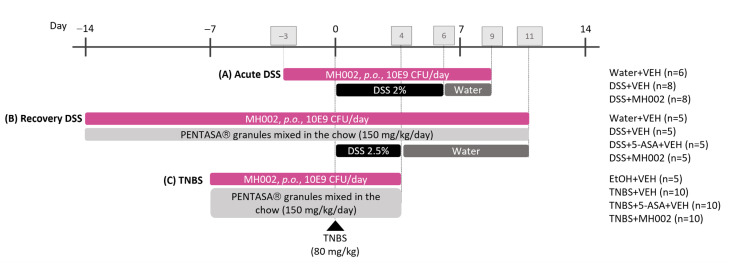
Experimental layout of the (**A**) acute dextran sodium sulfate (DSS)-, (**B**) recovery DSS-, and (**C**) 2,4,6-trinitrobenzenesulfonic acid (TNBS)-induced colitis studies. In the acute DSS, MH002 was administered to mice *p.o.* at the dose of 10E9 CFU/day for three days prior to DSS, during the seven days of DSS, and until the end of the study, i.e., for a total of 13 days. In the recovery study, MH002 was administered for two weeks prior to DSS in the same manner as in the acute study (*p.o.*, 10E9 CFU/day/dose), during the five days of DSS and until the end of the study, i.e., seven days after removal of DSS. In this study, MH002 was compared to 5-ASA (PENTASA^®^ granules mixed with rodent chow). Finally, the TNBS study was performed in rats, and colitis was induced by a single intrarectal injection of TNBS prepared in ethanol (EtOH). MH002 was administered seven days prior to TNBS injection, and until the end of the study, i.e., until four days after TNBS. Also in this study, MH002 was compared to 5-ASA, which was administered and prepared in the same manner as for the recovery DSS study (details in Section 4). Control groups, including the 5-ASA-treated animals received vehicle (VEH; phosphate-buffered saline) by intragastric gavage.

To validate the protective effects observed in vitro and ex vivo, MH002 was administered to mice and rats in a prophylactic setting, i.e., prior to the administration of dextran sodium sulfate (DSS) or 2,4,6-trinitrobenzenesulfonic acid (TNBS). The DSS model was performed in mice both in acute and recovery settings (Figure 7A,B), whereas TNBS was performed in an acute setting only (Figure 7C).

**Figure 8:**
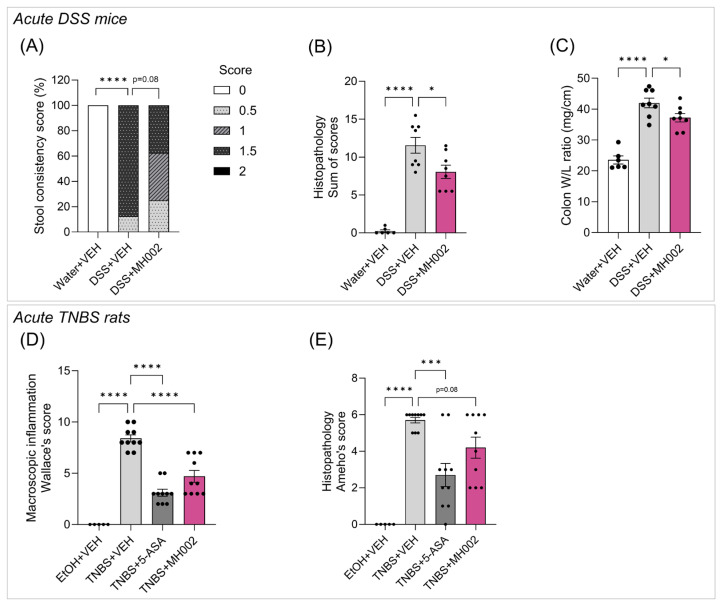
Effects of MH002 daily intake on acute colitis in mice and rats. (**A**) changes in stool consistency; (**B**) histopathology of the distal colon of mice; (**C**) colon weight/length (W/L) ratio; (**D**) macroscopic inflammation; and (**E**) histopathology of the distal colon of rats. Acute DSS in mice: in this study, MH002 was administered for three days prior to DSS, during the seven days of DSS, and until three days after DSS removal. Stools were evaluated as normal (=0), soft feces (=0.5), loose feces (=1), diarrhea (=1.5), and watery diarrhea (=2). Histopathology was calculated as a composite score for the presence of epithelial damage, inflammatory cells in the mucosa and submucosa, and ulcers (details in Supplementary Materials). Colon W/L was calculated as the ratio of the weight of the colon to its length (*n* = 6 for water + VEH, and *n* = 8 for DSS + VEH and DSS + MH002). TNBS in rats: in this study, colitis was induced by a single intrarectal injection of TNBS; MH002 was administered seven days prior to injection, and until four days after TNBS. Macroscopic inflammation was evaluated using the Wallace’s criteria as described in the Materials and Methods section, which scores the tissue according to the extent of hyperemia and ulceration. Histopathology was evaluated according to the criteria of Ameho as described in Supplementary Materials, which scores for the extent of epithelial erosion and ulceration, and for the presence of inflammatory infiltrates (*n* = 5 for EtOH + VEH, and *n* = 10 for TNBS + VEH, TNBS + 5-ASA, and TNBS + MH002). Data are presented as mean ± SE of the mean. Asterisks indicate significant difference *, *p* < 0.05; ***, *p* < 0.001; and ****, *p* < 0.0001 when compared to the disease (DSS or TNBS) group (one-way ANOVA with Dunnett’s post hoc test), and ns indicates not significant.

In both acute studies, pre-treatment with MH002 led to significant improvements locally, in the large intestine, and systemically, by reducing cytokine levels in the blood. More specifically, protective effects on stool consistency, histopathological scores, and colon weight/length (W/L) ratio, a measure of intestinal edema and inflammation, were observed in DSS-treated mice (Figure 8A–C).

In general, acute DSS-induced colitis results in intestinal epithelial damage, characterized by ulceration, bleeding, and diarrhea. In the MH002-treated group stool consistency scores improved at the end of the study, with 37.5% of the animals having a score of 1.5 (diarrhea) or 1 (loose feces), and 25% a score of 0.5 (soft feces). In contrast, in the DSS group, 87.5% and 0% of the animals showed a score of 1.5 or 1, respectively, and 12.5% a score of 0.5 (Figure 8A). At the microscopic level, histological examination of distal colon sections revealed sustained and significant inflammation and ulceration in the DSS + VEH-treated group when compared to non-DSS controls (*p* < 0.0001), i.e., DSS-treated animals showed an average composite score of 11.56. However, MH002 led to a significant histological improvement, displaying an average composite score of 8.06 (*p* = 0.015, Figure 8B). Finally, macroscopic evaluation of colonic inflammation was assessed by measuring the W/L ratio of the colon. Typically, DSS administration leads to an increase in the colon W/L as the colon thickens and shortens during inflammation. However, a significant decrease in colon W/L was observed in the DSS + MH002-treated group, representing an improvement in the inflammatory edema (*p* = 0.045, Figure 8C).

In a second study, a similar treatment approach was followed, but on a different rodent species. In colitic TNBS rats, protective effects were also observed both macroscopically and microscopically for MH002-treated animals (Figure 8D,E). The Wallace’s score, which evaluates ulceration and hyperemia at a macroscopic level, showed an average composite score of 8.4 in the TNBS + VEH group. In contrast, the TNBS + MH002-treated group showed an average score of 4.7, indicative of a significant (*p* < 0.0001) protection of the mucosa. This was comparable to TNBS + 5-ASA, which displayed an average composite score of 3.1 (Figure 8D). At the microscopic level, histological scores also improved, albeit not reaching statistical significance (Figure 8E and Figure S2).

**Figure 9:**
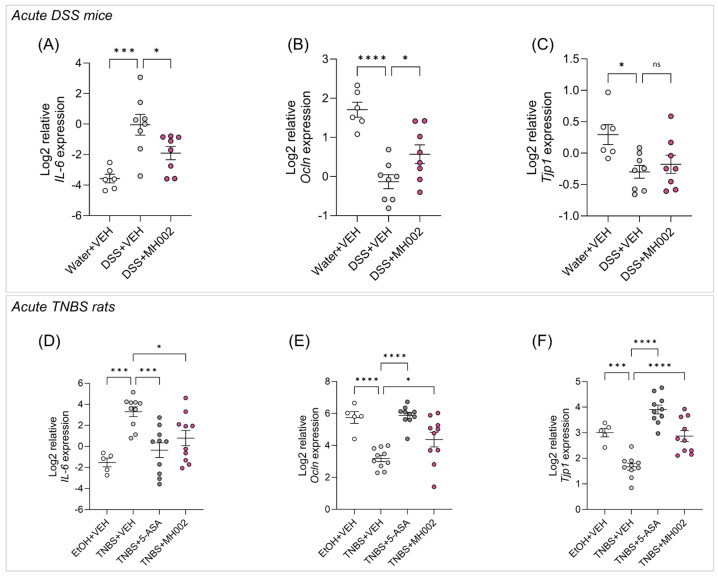
Effect of MH002 daily intake on the expression of inflammatory markers and tight junctions’ genes in the distal colon of acute colitic mice and rats. (**A**,**D**) interleukin 6 (*IL-6*); (**B**,**E**) occludin (*Ocln*), and (**C**,**F**) tight junction protein 1 (*Tjp1*). In DSS mice, *n* = 6 for water + VEH, and *n* = 8 for DSS + VEH and DSS + MH002. In TNBS rats, *n* = 5 for EtOH + VEH, and *n* = 10 for TNBS + VEH, TNBS + 5-ASA, and TNBS + MH002. Data are presented as mean ± SE of the mean. Asterisks indicate significant difference *, *p* < 0.05; ***, *p* < 0.001; and ****, *p* < 0.0001 when compared to the disease (DSS or TNBS) group (one-way ANOVA with Dunnett’s post hoc test), and ns indicates not significant.

Finally, in both studies, the expression of genes involved in barrier integrity, i.e., occludin (*Ocln*) and tight-junction protein 1 (*Tjp1*), encoding for zonula occludens 1 (ZO-1), in the distal colon was significantly increased in the MH002 (*Ocln*, *p* = 0.041 and *p* = 0.028 in mice and rats, respectively; *Tjp1*, *p* = 0.737 and *p* < 0.0001 in mice and rats, respectively) and 5-ASA (*Ocln* and *Tjp1*, *p* < 0.0001 in both mice and rats) groups when compared to the vehicle colitic controls, whereas a significant decrease in *IL-6*, a pro-inflammatory cytokine, was observed (MH002, *p* = 0.033 and *p* = 0.018 in mice and rats respectively; 5-ASA, *p* = 0.0005 in rats; Figure 9).

Finally, administration of either DSS or TNBS to animals resulted in elevated levels of circulating cytokines, indicative of systemic inflammation (Table 2). However, these were lowered upon MH002 and 5-ASA treatments in both studies, albeit not always reaching statistical significance (Table 2A,B). Despite these observed improvements, disease activity and body weight (BW) did not improve in either study, suggesting that in an acute setting, local changes driven by MH002 did not yet translate into a disease-modifying effect. Therefore, MH002 was further tested in a recovery DSS model.

### 2.6. Effect of MH002 on the Recovery from Acute DSS-Induced Colitis in Mice

**Figure 10:**
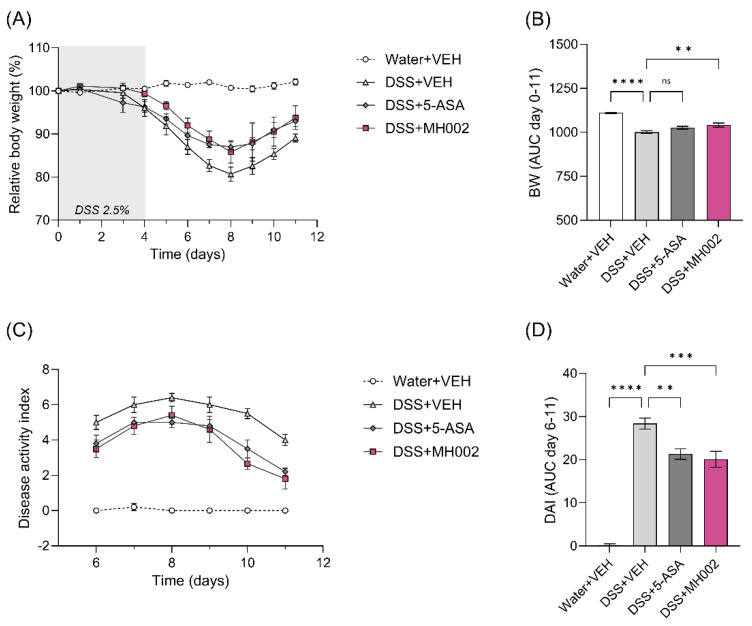
Effect of MH002 on the recovery from DSS-induced colitis in mice. In this study, MH002 was administered for two weeks prior to DSS, during the five days of DSS, and until seven days after DSS removal. (**A**,**B**) changes in body weight and (**C**,**D**) disease activity during the recovery phase of the disease. Body weight (BW) was followed daily, from day zero (start of DSS) until day eleven (termination). The area under the curve (AUC) was calculated during this period. The disease activity index (DAI) is a composite score combining body weight, stool consistency, and the presence of blood in stools (details in Section 4). This is presented after day six (two days after DSS removal), when systematic evaluation for the presence of blood in stools and stool consistency was performed. The AUC was also calculated for this period. Data are presented as mean ± SE of the mean (*n* = 5 animals for all groups). Asterisks indicate significant difference **, *p* < 0.01; ***, *p* < 0.001; and ****, *p* < 0.0001 when compared to the disease (DSS) group (one-way ANOVA with Dunnett’s post hoc test), and ns indicates not significant.

In this study, MH002 was administered for two weeks prior to DSS, after which DSS was administered for five days. The disease was monitored until sacrifice, i.e., seven days after removal of DSS (Figure 7B). Although BW gradually decreased in all DSS groups during the acute phase of the disease to then slightly recover, MH002 administration resulted in significantly (AUC, *p* = 0.009) lesser BW loss during the acute phase (Figure 10A,B), in a similar fashion as observed for the DSS + 5-ASA group.

**Figure 11:**
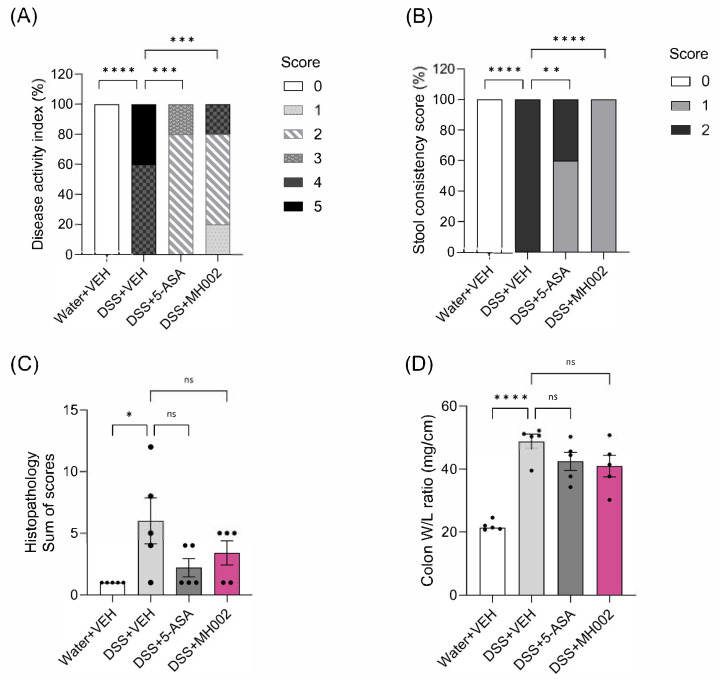
Effect of MH002 on the recovery from DSS-induced colitis in mice. In this study, MH002 was administered for two weeks prior to DSS, during the five days of DSS, and until seven days after DSS removal. (**A**) Changes in disease activity; (**B**) stool consistency; (**C**) histopathology of the distal colon; and (**D**) colon weight/length (W/L) ratio. The disease activity index (DAI) calculated at termination is a composite score combining body weight, stool consistency, and the presence of occult blood in the stool as determined by the hemoccult test (details in Section 4). For stool consistency, a score of 0 was assigned for well-formed pellets, 1 for pasty and semi-formed stools that did not adhere to the anus, and 2 for liquid stools that adhered to the anus. For occult blood, a score of 0 was assigned for no blood, and 1 for positive occult blood or for gross bleeding. Histopathology is a composite score that evaluates the extent of epithelial damage and severity of inflammation (details in Supplementary Materials). The colon W/L ratio was determined as described for the acute DSS study. (**C**,**D**) Data are presented as mean ± SE of the mean (*n* = 5 animals for all groups). Asterisks indicate significant difference *, *p* < 0.05; **, *p* < 0.01; ***, *p* < 0.001; and ****, *p* < 0.0001 when compared to the disease (DSS) group (one-way ANOVA with Dunnett’s post hoc test), and ns indicates not significant.

Disease activity, which is calculated based on the percentage of BW loss, stool consistency, and presence of blood in the stools, increased in the DSS + VEH-treated animals during the peak of disease (i.e., four days after DSS removal), to then slowly recover. In contrast, DSS + MH002-treated mice showed lower disease activity over time, with the most pronounced effects noticed 5–7 days after DSS removal (Figure 10C,D). Indeed, the area under the curve (AUC) between days 6 and 11 showed a significant improvement in the MH002-treated group (*p* = 0.0008). The effects were comparable to 5-ASA (*p* = 0.003). In addition, at the end of the study (i.e., seven days after DSS removal), notable protective effects were observed in the DSS + MH002 group, when compared to DSS + VEH-treated animals, namely on stool consistency (*p* < 0.0001) and final disease activity (*p* = 0.0002), when the composite score included the presence of occult blood (Figure 11A,B). More specifically, whereas in the DSS + VEH group animals had either a score of 4 (60%) or 5 (40%) for disease activity, this was reduced in the DSS + MH002-treated group to 20% and 0%, respectively (Figure 11A). In addition, most animals (60%) had a score of 2 in the DSS + MH002-treated group, and 20% had a score of 1. Improvement in stool consistency was also notorious, with 100% of the DSS + MH002-treated animals having a score of 1 (soft feces) at the end of the study, whereas in the DSS + VEH group, all animals had a score of 2 (diarrhea) (Figure 11B).

Finally, despite a lack of statistical significance, MH002 showed a trend to improve histopathology scores and colon W/L ratio to a similar extent as observed for the 5-ASA-treated group (Figure 11C,D and Figure S3). Altogether, these results suggest that MH002 improved local intestinal inflammation during the acute phase of the disease, which later translated into a faster disease recovery, and lower systemic levels of inflammatory mediators (Table 2C).

## 3. Discussion

Microbiome-based interventions are recognized as valid therapeutic strategies that have the potential to induce or maintain remission in IBD patients [32,33]. In the current study, we have characterized MH002, a six-strain consortium for its anti-inflammatory and mucosal healing effects. This consortium of bacteria has been assembled with the aim of increasing butyrate levels in the gut and thereby conferring local health benefits. Butyrate has an important role in gut health, by providing support to colonocyte function, decreasing inflammation, maintaining the intestinal barrier, and promoting a healthy microbiome (reviewed in [15]). Low levels of butyrate and butyrate producers are common features of numerous gastrointestinal diseases, and in this context, butyrate has been shown to be protective not only in IBD [34,35], but also in graft-versus-host disease [36,37], and colorectal cancer [38].

The ability of MH002 to produce butyrate was confirmed in fed-batch cultures in vitro, where high levels (>30 mM) were rapidly reached under colon-like conditions. Albeit not as high, under dysbiotic conditions, in the presence of an IBD-microbial stool background, levels also rapidly increased, reaching concentrations of about 16 mM. This is of relevance as MH002 will be orally delivered to the intestine of IBD patients and potential therapeutic effects will depend on either its capacity to reduce inflammation and promote intestinal barrier function in the presence of a resident microbiome, and/or the capacity to alter the gut microbial environment and improve host–microbe interactions. Nevertheless, butyrate concentrations in the SHIME^®^ gradually returned to control levels, thereby suggesting that effects are transient and most likely repeated daily dosing will be necessary to maintain the protective effects of MH002 in patients. Nonetheless, whether tested directly, or indirectly in the presence of a fecal microbiome, we have shown that MH002 improves intestinal epithelial barrier integrity and cytokine release in a co-culture model of intestinal epithelial cells (Caco-2) with activated immune cells (ConA-stimulated PBMCs). The observed effects were confirmed upon ‘treatment’ of IBD stool samples with MH002 in the SHIME^®^ experiment. This and similar models, which mimic the gut epithelium/immune cell interface, are useful in the search for drugs or food substances that can be used to treat or prevent IBD-like symptoms [36], for instance, butyrate has been shown to protect against barrier disruption in a Caco-2/PBMCs co-culture model with lipopolysaccharide- (LPS) and anti-CD3/CD28-activated PBMCs [37]. The results presented here confirm this: not only does butyrate protect the barrier integrity of intestinal epithelial cell monolayers but it also reduces pro-inflammatory cytokine release in Caco-2/ConA-activated PBMCs co-cultures. Positive effects were also observed in a wound healing assay performed in colonocyte-like (T84) cells, once more confirming MH002 protective effects on the intestinal epithelium. Of importance, sodium butyrate has been shown by others to have similar effects in small intestinal epithelial cells in vitro [39]. Indeed, our results confirm the protective effects of butyrate also in colonocyte-like cells, although this is dose-dependent. Nevertheless, and in general, 5 mM butyrate, which represents physiological levels present in the colon of healthy individuals, mimics MH002 protective effects in vitro.

To elucidate and confirm the mechanisms by which MH002 can improve disease, biopsies were collected from IBD patients and used for either organoid preparation or for colonic explants. MH002 was shown to increase the TEER of the monolayers when compared to patient-matched cells treated with either cell culture medium or lyoprotectant. In addition, the expression of *OCLN* significantly increased upon MH002 treatment. Occludin is a tight junction protein that promotes epithelial cell adhesion. It binds to ZO-1 to limit paracellular permeability to macromolecules [40,41,42,43], and its expression has been shown to be reduced in mucosal samples from both CD and UC patients [44,45,46]. In contrast, we observed a trend to reduce the expression of *CLDN-2* in MH002-treated cells, when compared to patient-matched organoids that were treated with either a cell culture medium or lyoprotectant. This is an important pore-forming claudin [47] that increases barrier permeability [48] and has been shown to be elevated in the mucosa of IBD patients [45,49,50,51], and to possibly contribute to neoplasia [51]. Finally, *MUC-2* expression showed a tendency to increase upon MH002 treatment. MUC-2 is a gel-forming mucin produced by goblet cells, and it is the major component of the intestinal mucin layer [52]. It has been described to be induced by toll-like receptor (TLR) ligands, and interestingly, *Roseburia* spp. have been shown to upregulate *Ocln* and *Muc-2* in the intestine of ethanol-fed mice, possibly via flagellin recognition by TLR5, thereby promoting barrier function [53]. Despite these benefits, the protective effect of MH002 on the expression of tight junction genes in patients-derived organoids may be a specific one, as for instance, the expression of *TJP1*, encoding ZO-1, was unaltered. In contrast, in the distal colon of mice and rats, *Tjp1* was found to be upregulated by MH002 treatment, an effect also observed by others upon butyrate treatment [54], which together with *Ocln*, suggests either a species-specific effect, a differential effect under inflamed conditions in the colitic animals, or the use of MH002 product strains in vivo vs. produced metabolites as tested in the organoids. The biopsies collected from IBD patients were also treated ex vivo with MH002-derived metabolites and compared to either medium or lyoprotectant. In addition, explants were simultaneously stimulated with human anti-CD3 to increase the secretion of T cell cytokines. Although several cytokines were measured after 24 h, only IL-17 and IL-22 were detected at relevant levels. This contrasts with published work from Vadstrup and colleagues [31], which were able to measure several cytokines in a similar experiment, most likely because in the current study, we have tested biopsies collected from non-inflamed mucosal areas. Nonetheless, MH002 was shown to reduce both IL-17 and IL-22 upon anti-CD3 treatment, thereby suggesting a protective effect on T cell-mediated cytokine secretion, particularly cytokines described to be produced by T helper (Th)17 cells. These cells are important to maintain gut mucosal immune homeostasis by secreting small quantities of IL-17 and IL-22. This promotes epithelial cell proliferation and upregulation of antimicrobial peptides, particularly through IL-22, thereby protecting the intestinal mucosa from pathogenic infections, and preserving an intact epithelium. However, when left unchecked, the proliferation of Th17 cells contributes to disease progression, particularly via IL-17-mediated fibrinogenic actions [55]. The MH002-induced decrease in IL-17 was confirmed in Caco-2/PBMCs co-cultures that were stimulated with ConA. In contrast, in Caco-2/PBMCs, IL-22 increased upon treatment with MH002 cell-free supernatant but showed an IBD stool background-dependent effect in the IBD-M-SHIME^®^ experiment, thereby suggesting either cell-specific effects (local vs. periphery) or a distinct regulation of IL-22 by microbiome-derived products. In the gut, IL-22 is under the transcriptional control of the aryl hydrocarbon receptor (AhR) [56], a master regulator of intestinal homeostasis [57]. Even though AhR is mostly recognized to be activated by tryptophan-derived microbial metabolites, it is also activated by butyrate [58]. On the other hand, stools collected from IBD patients were shown to have reduced levels of tryptophan metabolites and AhR activation, in a Caspase recruitment domain family member 9 (CARD9) genotype-dependent manner [58]. Therefore, it is conceivable that the effects observed in the SHIME^®^ are controlled by other mechanisms than butyrate only, and in an IBD-stool-dependent manner [59], an effect which is then also regulated differently in the absence of a fecal microbiome in PBMCs and in colonic explants. In Caco-2/PBMCs co-cultures, other cytokines than IL-17 were found to be reduced by MH002 cell-free supernatant. These included IL-21 (produced by Th17 cells, among others), a cytokine that has numerous effects, including differentiation of T follicular helper cells, inhibition of regulatory T cells, and production of autoantibodies [60]. In addition, IL-4 and IL-9 were found to be reduced by MH002 cell-free supernatant, thus suggesting a reduction in cytokines produced by other T cell populations, such as Th2 and Th9, respectively. This could imply a reduction in type 2 immune responses [61].

In animals, namely on models of chemical-induced colitis, MH002 was also found to be protective. Although in an acute setting MH002 treatment did not result in improved body weight or disease activity, which combines body weight loss, fecal blood loss, and stool consistency, several relevant endpoints were found to improve, namely histopathological changes, macroscopic colonic edema and inflammation, and stool consistency, i.e., improvement of diarrhea. This was observed both in acute DSS colitis, which is mostly driven by innate immunity, and in TNBS-induced colitis, where mucosal damage is rather associated with T cell reactivity [62]. These local colonic improvements were accompanied by reduced local inflammation, as determined by the reduced expression levels of *IL-6* in the colon. DSS and TNBS elicit distinct blood cytokine profiles, with acute DSS being characterized by the elevation of IL-6, C-X-C motif chemokine ligand (Cxcl)1, Tnfα, and IL-17, whereas TNBS has rather a Th1/Th17 profile with elevated levels of IL-12, IL-17, and Ifnγ [63]. We have observed that indeed, treatment with MH002 resulted in reduced levels of systemic cytokines, such as IL-6, Cxcl1, and granulocyte colony stimulating factor (G-CSF) in DSS-treated animals, and IL-2, Tnfα, and Ifnγ in TNBS rats. This suggests that reduction of local inflammation and improved mucosal damage resulted in lower systemic levels of inflammatory mediators in both studies. Because bacterial strains could require some time to thrive in the colon, particularly under such acute conditions, we hypothesized that MH002 benefits on body weight or disease activity would occur during the later stages of the disease. For that, we have tested MH002 in a model of epithelial recovery, where C57BL/6 mice, upon removal of DSS from the drinking water, are described to transition from an acute-to-chronic state, where the disease progresses towards mucosal healing [64]. Indeed, in C57BL/6 mice, after short exposure to DSS, the disease progresses to a chronic-like phase, where despite the presence of milder symptoms, the disease does not fully resolve. This contrasts with other strains of mice, in which upon acute exposure to DSS, the disease completely resolves shortly after DSS removal. Therefore, the use of C57BL/6 mice allows studying disease during the acute-to-chronic transition phase, with progressive healing of the intestinal epithelium [64]. Therefore, in this model, disease parameters were evaluated during and after the peak of disease, which normally occurs 3–5 days after DSS removal [64]. Indeed, 4–5 days after DSS had been removed from the drinking water, the effects of MH002 became notorious on body weight and disease activity, in a way comparable to 5-ASA. This was accompanied by improved histopathological and stool consistency scores, final disease activity, and colon weight-to-length ratio. Altogether, these results suggest that MH002 is a promising microbiome-based therapeutic option for IBD, as it can protect the intestinal barrier, by modulating the expression of specific tight junctions’ genes described to regulate intestinal permeability. In addition, it improves local and systemic inflammation, thereby resulting in an overall disease amelioration, with concomitant mucosal healing. However, it remains to be evaluated whether MH002 can also improve response to treatment, such as biologics.

## 4. Materials and Methods

### 4.1. MH002 Test Materials

MH002 consists of six commensal bacterial strains, optimized into a miniature ecosystem using a proprietary technology platform. The strains belong to six different genera, including MH002 strain 01 (MH002-01) *Faecalibacterium* sp., MH002-02 *Anaerostipes* sp., MH002-03 *Butyricicoccus pullicaecorum* 25-3T [65], MH002-04 *Akkermansia* sp., MH002-05 *Roseburia* sp., and MH002-06 *Lactiplantibacillus* sp. All strains have been deposited at the Belgian culture collection BCCM/LMG, Ghent.

#### 4.1.1. Fed-Batch Cultures

To prepare samples for in vitro cell cultures, lyophilized MH002 was added at a final concentration of 0.8–1.6 mg/mL to bioreactors containing nutritional medium (composition in [66]). MH002 was kept in culture for seven days under anaerobic and pH-controlled conditions. Samples were collected at regular intervals to measure short-chain fatty acids (SCFAs), and at day four for evaluation in in vitro cell cultures. For that, samples were first centrifuged to pellet the bacteria (5340× *g*; 10 min), and the remaining supernatant was collected, sterile-filtered (0.22 µm), aliquoted, and stored at −80 °C until further use (herein referred to as cell-free supernatant). For ex vivo experiments, a freeze-dried control carrier (mainly lyoprotectant) was inoculated in the same manner and incubated in parallel under the same conditions.

#### 4.1.2. Formulations for In Vivo Studies

In all in vivo studies, MH002 was administered daily (1x/day) at the dose of 10E9 colony-forming units (CFU)/dose/day by oral gavage. In the mouse dextran sodium sulfate (DSS) studies, MH002 was administered as an active culture. For that, MH002 was prepared from a lyophilized product and cultured in bioreactors under anaerobic and pH-controlled conditions as described above. On each dosing day, MH002 was harvested under anaerobic conditions, washed in 1X Dulbecco’s Phosphate-Buffered Saline (D-PBS), concentrated by centrifugation (5340× *g*; 10 min), and resuspended in 1X D-PBS. In the 2,4,6-trinitrobenzenesulfonic acid (TNBS) rat study, MH002 was administered as lyophilized powder reconstituted in 1X D-PBS. For that, lyophilized MH002 was washed in 1X D-PBS, concentrated by centrifugation (5340× *g*; 10 min), and resuspended in 1X D-PBS. All test items were kept under anaerobic conditions and at ambient temperature until intragastric gavage, which was performed within four hours of test item preparation.

### 4.2. Caco-2/PBMCs Co-Cultures

Caco-2 cells (HTB-37, ATCC), between passages 44 and 65, were seeded in 24-well semi-permeable inserts (0.4 μm pore size PET membrane; Millicell^®^ inserts, Merck-Millipore, Hoeilaart, Belgium). Cells were cultured and resuspended in Dulbecco’s Modified Eagle Medium (DMEM) containing 4.5 g/L glucose (Sigma-Aldrich, Hoeilaart, Belgium) and supplemented with 20% heat-inactivated fetal bovine serum, 10 mM HEPES, and 1% penicillin/streptomycin (all from Gibco, Thermo Fisher Scientific, Dilbeek, Belgium) (complete medium). Cells were seeded on the apical side of the inserts at the density of 1 × 10^5^ cells/insert. The basolateral chamber was filled with a complete medium. Caco-2 cells were allowed to differentiate into a functional monolayer for 14–16 days at 37 °C in a humidified atmosphere of air/CO_2_ (95%/5%), and the medium was replenished three times per week.

After 14–16 days, the transepithelial electrical resistance (TEER) was measured by using a Millicell^®^ ERS-2 Voltohmmeter (Merck-Millipore, Burlington, MA, USA), and corrected with the resistance value of an empty insert. Final TEER values were calculated by multiplying the resistance by the area of the insert (0.33 cm^2^). Then, all inserts were transferred to a separate 24-well plate containing human peripheral blood mononuclear cells (PBMCs). Briefly, PBMCs were isolated from buffy coats obtained from healthy-donor blood donations at the Red Cross (Ghent, Belgium). PBMCs were isolated by following the protocol as described by Kleiveland (2015) in Verhoeckx and colleagues [67] with some modifications. Mononuclear cells were obtained by using density gradient centrifugation and Lymphoprep™ (Stem Cell Technologies, Saint Égrève, France) and frozen in 90% heat-inactivated fetal bovine serum (Gibco, Thermo Fisher Scientific, Dilbeek, Belgium) and 10% DMSO (Sigma-Aldrich, Hoeilaart, Belgium) until further use. On the day of the experiment, cells were thawed, resuspended in a complete medium, and seeded in 24-well plates at the density of 1x10^6^ cells/well. Upon seeding, cells were simultaneously stimulated with 10 μg/mL concanavalin A (ConA; InvivoGen, Toulouse, France). After transfer of the Caco-2-bearing inserts into PBMCs-containing plates, the apical supernatant was removed from the inserts and the Caco-2 cells were apically treated with either MH002 cell-free supernatant 5-fold diluted (*v*/*v*) in complete medium or 5 mM sodium butyrate (Sigma-Aldrich, Hoeilaart, Belgium) prepared in the same medium. All treatments were prepared in complete medium and tested in triplicate. Cells were incubated for 48 h at 37 °C in a humidified atmosphere of air/CO_2_ (95%/5%), after which the TEER was once more measured and normalized to the baseline value after blank correction. At this point, the basolateral cell supernatants were also collected for cytokine measurement. Interleukin (IL)-4, IL 9, IL-17A, IL-21, and IL-22 were quantified by a Luminex^®^ magnetic beads-based assay (eBioscience, Thermo Fisher Scientific, Dilbeek, Belgium) according to the manufacturer’s instructions. Luminex^®^ data was recorded in a MAGPIX Instrument (Luminex Corporation, Austin, TX, USA) and analyzed using the ProcartaPlex Analysis App software from Thermo Fisher Scientific (v1.0, Thermo Fisher Scientific, Waltham, MA, USA). The release of lactate dehydrogenase (LDH), a marker of cell death, was measured on the basolateral supernatants using the Lactate Dehydrogenase Activity Colorimetric Assay Kit (Sigma-Aldrich, Hoeilaart, Belgium), according to the manufacturer’s instructions.

### 4.3. Wound Healing Assay (WHA)

T84 cells (ECACC, Sigma-Aldrich, Hoeilaart, Belgium) were cultured and resuspended in DMEM:F12 supplemented with 5% heat-inactivated fetal bovine serum and 1% penicillin/streptomycin (all from Gibco, Thermo Fisher Scientific, Dilbeek, Belgium). Cells at passages 21 and 22 were seeded in 24-well plates at a density of 5 × 10^5^ cells/well and incubated at 37 °C in a humidified atmosphere of air/CO_2_ (95%/5%) for seven days, after which a manual ‘scratch’ was made in all wells with the help of a 10 μL sterile tip. Then, the wells were washed twice with sterile D-PBS and subsequently treated with either MH002 cell-free supernatant 5- or 7-fold diluted (*v*/*v*) in serum-free cell culture medium or sodium butyrate for comparison purposes. Pictures were collected at this point for baseline correction. Cells were treated for 24 or 48 h hours at 37 °C in a humidified atmosphere of air/CO_2_ (95%/5%), after which pictures were collected. Pictures were taken with the help of the Cytation 5 Cell Imaging Multi-Mode Reader (Agilent BioTek, Santa Clara, CA, USA), and the wound area was measured by using the MRI Wound Healing Tool macro for ImageJ/Fiji^®^ v1.49 [68]. Wound closure was calculated as the relative percentage of the baseline wound area. All conditions were tested in triplicate.

### 4.4. Simulator of the Human Intestinal Microbial Ecosystem (SHIME^®^)

To simulate an IBD-like colonic environment the SHIME^®^, a well-described in vitro system that mimics the different regions of the human gut in a succession of five reactors [69,70] was used. For this, fecal slurries of six patients with active UC (four males and two females, aged 25–60 years) were inoculated in a standardized in vitro gut model (IBD-M-SHIME^®^) [66] with some modifications. Briefly, fecal slurries were homogenized with an equal volume of cryoprotectant solution (modified from Hoefman and colleagues [71]) under anoxic conditions, snap-frozen in liquid nitrogen, and stored at −80 °C until further use. After thawing, the cryoprotectant was removed by centrifugation (6000× *g*; 10 min) and replaced with 0.5 vol. 1X D-PBS. The fecal slurries were incubated (5% (*v*/*v*); 12–16 h; 37 °C; pH 6.15–6.35) prior to use as described in [66]. For each treatment, the same concentration of total bacteria was used (approx. 1 × 10^8^ bacteria/mL). A total of 24 simulated proximal colon environments (i.e., two test items/donor and two replicates/test item) were run for eight days, with a single dose administration of either MH002 or control (1X D-PBS) at day zero. For that, MH002 was prepared as described above, harvested under anaerobic conditions, concentrated by centrifugation (5340× *g*; 10 min), and resuspended in 1X D-PBS prior to addition to the SHIME^®^ reactors. During the eight days, samples were collected at regular intervals for SCFA measurement. For in vitro cell cultures, samples were collected after 24 h and tested as described above. Sodium butyrate (2.5 and 5 mM) was also tested for comparison purposes on in vitro cell cultures. This study was conducted in accordance with the Declaration of Helsinki, approved by the Ethics Committee of the University Hospital Ghent (B670201836585), and all volunteers received and signed an informed consent form.

### 4.5. SCFA Measurement

All samples collected from reactors’ experiments were used to measure SCFA levels, namely, acetate, propionate, and butyrate, by gas chromatography (with flame ionization detection) as previously described [66].

### 4.6. Biopsy-Derived Intestinal Organoids and Colonic Explants from IBD Patients

#### 4.6.1. Biopsy Collection

Mucosal biopsies from non-inflamed areas of the colon of active IBD patients of both genders, undergoing routine endoscopy for follow-up on disease status, were sampled during endoscopy at the University Hospital of Leuven (UZ Leuven, Leuven, Belgium). Patients were enrolled according to the following inclusion criteria: (i) diagnosis of ulcerative colitis or Crohn’s disease with colonic disease; (ii) presence of active disease confirmed by endoscopy; (iii) no signs of infection at time of endoscopy; (iv) age between 18 and 80 years old; (v) no pregnancy; (vi) hepatitis C- B- and HIV-negative; and (vii) stable use of concomitant medication 30 days prior to the study. Whenever possible, 10 biopsies per patient were collected from non-inflamed colonic areas to allow organoid preparation from stem cells in the crypts. Biopsies were used for the preparation of intestinal organoids and/or colonic explants. Ethical approval was provided by the Ethics Board of the University Hospital Leuven and all patients provided written informed consent.

#### 4.6.2. Preparation of Intestinal Organoids

For the preparation of intestinal organoids, four biopsies from macroscopically non-inflamed colonic areas were collected in DMEM containing high glucose and GlutaMaxTM (Gibco, Thermo Fisher Scientific, Dilbeek, Belgium) and supplemented with 10% heat-inactivated fetal bovine serum, 1% non-essential amino acids, 1% sodium pyruvate, 1% penicillin/streptomycin, and 50 μg/mL gentamicin (all from Gibco, Thermo Fisher Scientific, Dilbeek, Belgium) and immediately placed at 4 °C for transport (on ice) until epithelial isolation was performed. Intestinal crypts were isolated as previously described by Vanhove and colleagues [72], and at the end of the procedure they were resuspended in Matrigel (Growth Factor Reduced, phenol-red-free, Corning, NY, USA) diluted with basal medium (1:1). Wells were filled with complete human expansion medium (full list of components in Vanhove and colleagues [72]), which was replenished every-other-day to allow organoids growth. During the ensuing 7–10 days, the organoids were split and expanded, to be finally transferred to the apical compartment of 24-well semi-permeable inserts (6.5 mm Transwells^®^, 0.4 µm pore size PET membrane, Costar^®^, Corning, Willebroek, Belgium) at the density of 1 × 10^6^ cells/insert. The procedure to form 2D epithelial cell monolayers on membrane inserts from organoids was based on the protocol described by Vancamelbeke and colleagues [29]. Briefly, cells were pre-incubated with 10 µM Rho associated kinase inhibitor (ROCKi, Selleckchem, Munich, Germany) to maintain their pluripotent state. The basolateral compartment of the wells was filled with a complete human expansion medium containing 10 µM ROCKi. Cell-containing inserts were incubated at 37 °C in a humidified incubator (95%/5% air/CO_2_) to allow attachment. After 24 h, the medium from the apical and basolateral compartments was replaced with a complete human expansion medium without ROCKi. This medium change was performed every-other-day until confluent and polarized monolayers were formed (5–9 days post-seeding). The functional integrity of the monolayers was monitored regularly by measuring the TEER using the EVOM2 epithelial Volt/Ohm meter and STX2 chopstick electrode set (World Precision Instruments, Sarasota, FL, USA), and corrected with the resistance value of an empty insert. Final TEER values were calculated by multiplying the resistance by the area of the insert. Once the TEER values reached levels >250 Ω.cm^2^, the monolayers were considered polarized and functionally intact and were apically treated with human expansion medium (Med), MH002 cell-free supernatant, or lyoprotectant (lyoprt)-supernatant 7-fold diluted (*v*/*v*) in human expansion medium. The basolateral compartment was filled with human expansion medium. Cells were treated for a total of 48 h, and the TEER was measured after 24 and 48 h; results are shown as % of baseline. Each condition was tested in duplicate whenever possible. After 48 h treatment, cells were washed once with 1X D-PBS, homogenized in RLT lysis buffer (RNeasy mini kit, Qiagen, Hilden, Germany) and stored at −80 °C until RNA extraction and used in real-time (RT) quantitative (q)PCR (RT-qPCR) as described in Supplementary Materials.

#### 4.6.3. Preparation of Colonic Explants

Six biopsies from macroscopically non-inflamed colonic areas were collected in Roswell Park Memorial Institute (RPMI) supplemented with 10% heat-inactivated fetal bovine serum, 1% non-essential amino acids, 1% sodium pyruvate, 1% penicillin/streptomycin, 200 µg/mL gentamycin (all from Gibco, Thermo Fisher Scientific, Dilbeek, Belgium), and 2.5 µg/mL amphotericin B (Sigma-Aldrich, Hoeilaart, Belgium) (explant medium) and immediately placed at 4 °C. Biopsies were thoroughly washed and divided over six wells of a 24-well plate. Then, all wells were stimulated with 10 µg/mL human anti-CD3 (MA1-10175, anti-CD3e monoclonal antibody clone OKT3; Thermo Fischer Scientific, Dilbeek, Belgium), prepared in an explant medium. Simultaneously, biopsies were treated with explant medium (Med), MH002 cell-free supernatant, or lyoprt-supernatant 7-fold diluted (*v*/*v*) in explant medium. Each condition was tested in duplicate whenever possible. Biopsies were treated for 24 h in aerobic conditions (37 °C; 95%/5% air/CO_2_). After that, the cell culture supernatant was collected for cytokine and LDH activity measurements. The levels of interleukin (IL)-4, IL 9, IL-12p70, IL-17A, IL-21, and IL-22 were quantified by a Luminex^®^ magnetic beads-based assay, and interferon (IFN)γ by enzyme-linked immunosorbent assay (ELISA) (both from eBioscience, Thermo Fisher Scientific, Dilbeek, Belgium), according to the manufacturer’s instructions. Luminex^®^ data was recorded in a MAGPIX Instrument (Luminex Corporation, Austin, TX, USA) and analyzed as previously described. The release of LDH was measured as described above.

### 4.7. Chemical-Induced Colitis in Rats and Mice

MH002 was tested in three rodent models of chemical-induced colitis: in mice, MH002 was tested in models of acute or recovery DSS-induced colitis; in rats, MH002 was tested in the TNBS-induced acute colitis model. For the acute DSS colitis study, 8-week-old C57BL/6J male mice were obtained from Harlan Laboratories (Horst, The Netherlands), and maintained under standard laboratory conditions with ad libitum access to food (standard rodent chow, Carfil Labofood, Pavan Service, Belgium) and water. To homogenize the microflora, mice were co-housed before randomization for one week. After that, animals were divided into three groups: water + VEH (*n* = 6), DSS + VEH (*n* = 8), or DSS + MH002 (*n* = 8). MH002 was administered three days prior to DSS and until the end of the study. To induce colitis, 2% (*w*/*v*) DSS (molecular weight 36,000–50,000, MB Biomedicals, Illkirch, France) was administered in the drinking water for seven days, followed by regular water for three days. The water + VEH group received normal drinking water throughout the experiment. Body weight and disease activity were recorded daily. A disease activity index (DAI) was calculated as the composite score of body weight loss (0, <5%; 1, 5–10%, 2, 10–15%, 3, 15 20%, 4, >20%), stool consistency (0, normal; 0.5, soft feces; 1, loose feces; 1.5, diarrhea; 2, watery diarrhea), and detection of occult blood in feces (0, none; 1, hemoccult positive; 2, gross bleeding). Occult blood was detected using the ColoScreen Hemoccult kit (Helena Laboratories Inc., Beaumont, Texas, USA). Ten days after DSS, mice were anesthetized, and blood was collected from the retro-orbital sinus. Blood was allowed to clot and then centrifuged (10,000 rpm; 10 min) for collection of serum for cytokine measurement (as described in Supplementary Materials). Mice were then euthanized by cervical dislocation; the colon was removed, and weight and length were measured. Segments of the distal colon were sectioned, rinsed with PBX 1X, and stored in RNA*later*^®^ (Thermo Fisher Scientific, Dilbeek, Belgium) for RNA isolation, or in fixative solution for histological preparations (see details in Supplementary Materials). This study was approved by the Institutional Review Board of the Faculty of Medicine and Health Science of Ghent University.

The TNBS and recovery DSS studies were performed by IBD-Biotech (Faculty of Medicine, Lille, France). For the TNBS study, 5-week-old male Sprague Dawley rats were obtained from Janvier Laboratories (Le Genest Saint Isle, France), and maintained under standard laboratory conditions with ad libitum access to food (standard rodent chow, SAFE, Safe A04 P2,5) and water. After one week of acclimatization, animals were randomized and allocated to four groups: EtOH + VEH (*n* = 5), TNBS + VEH (*n* = 10), TNBS + 5-ASA (*n* = 10), and TNBS + MH002 (*n* = 10). MH002 was administered seven days prior to TNBS, and until the end of the study. In this study, the effects of MH002 were compared to 5-ASA. For that, PENTASA^®^ granules (Ferring, Hoofddorp, The Netherlands) were administered mixed with the rodent chow (150 mg/kg body weight). To induce colitis, rats were anesthetized by subcutaneous injection of xylazine (12.5 mg/kg; Rompun 2%; Bayer, Loos, France) and ketamin (25 mg/kg; Ketamin 1000; Virbac, Carros, France). Colitis was induced by intrarectal injection of 250 μL TNBS (Sigma Aldrich, St. Quentin Fallavier, France) at the dose of 80 mg/kg prepared in 40% ethanol (EtOH). Body weight was recorded throughout the colitis period, and four days after TNBS administration, animals were euthanized by cervical dislocation. At euthanasia, blood was collected by cardiac puncture, allowed to clot, and centrifuged (2300× *g*; 10 min) for collection of serum for cytokine measurement (as described in Supplementary Materials). The colon was collected and immediately examined for the presence of macroscopic lesions by using the Wallace score rates [73]. Briefly, on a scale from 0 to 10, the Wallace criteria examine features of inflammation, such as hyperemia, thickening of the bowel, and extent of ulceration (0, no inflammation; 1, hyperemia without ulcerations; 2, hyperemia with thickening of the mucosa without ulcerations; 3, one ulceration without thickening of the colonic wall; 4, two or more ulcerative or inflammatory sites; 5, two or more ulcerative and inflammatory sites with an extent > 1 cm; 6, ulcerative or inflammatory site > 2 cm; 7, ulcerative or inflammatory site > 3 cm; 8, ulcerative or inflammatory site > 4 cm; 9, ulcerative or inflammatory site > 5 cm; 10, ulcerative or inflammatory site > 6 cm). After macroscopic examination, a colon specimen located 2 cm above the anal canal was used for histological evaluation (details in Supplementary Materials). A sample from the distal colon was also collected in RNA*later*^®^ (Thermo Fisher Scientific, Illkirch, France) for RNA isolation (see Supplementary Materials).

For the recovery DSS study, 9-week-old male C57BL/6 mice were obtained from Charles River Laboratories (L’Arbresle, France), and maintained under standard laboratory conditions with ad libitum access to food (standard rodent chow) and water. After one week of acclimatization, animals were randomized and allocated to four groups: water + VEH (*n* = 5), DSS + VEH (*n* = 5), DSS + 5-ASA (*n* = 5), and DSS + MH002 (*n* = 5). MH002 was administered 14 days prior to the induction of colitis, and until the end of the study. In this study, the effects of MH002 were also compared to PENTASA^®^ (Ferring, Hoofddorp, The Netherlands), which was administered mixed with the rodent chow, similarly to what was carried out for the TNBS rats. To induce colitis, 2.5% (*w*/*v*) DSS (molecular weight 45,000, TDB Consultancy AB, Uppsala, Sweden) was administered in the drinking water for five days, followed by regular water for seven days. The water + VEH group received normal drinking water throughout the experiment. Body weight was recorded daily, and disease activity was recorded systematically from two days after DSS removal (experimental days 6 to 11). The DAI was calculated as the composite score of body weight loss (0, <5%; 1, 5–10%, 2, 10–15%, 3, 15–20%, 4, >20%), stool consistency (0, normal; 1, soft feces; 2, diarrhea; 3, watery diarrhea), and detection of blood in feces (0, absence; 1, presence). At the end of the study, i.e., 12 days after DSS, animals were euthanized by cervical dislocation. At euthanasia, a different DAI was calculated which, in addition to body weight loss as described above, considered the presence of occult blood recorded using the hemoccult method (0, negative; 1, positive occult blood or gross bleeding), and a 0–2 scale for stool consistency (0, well-formed pellets; 1, semi-formed stools that did not adhere to the anus; 2, liquid stools that adhered to the anus). Then, blood was collected by cardiac puncture, allowed to clot, and centrifuged (2300× *g*; 10 min) for collection of serum for cytokine measurement (as described in Supplementary Materials). At euthanasia, the colon was dissected, and the length was recorded. The luminal content was then removed before weighing the colon to determine its weight/length (W/L) ratio. Samples from the distal colon were also collected in a fixative solution for histology (see Supplementary Materials). All animal experiments conducted at IBD Biotech were performed according to French governmental guidelines (articles R214-87 à R214-137 code rural update 13 February 2013 according to European directive 2010/63/UE) and those of the Nord-Pas de Calais Ethical Committee for animal use.

In all studies, the control groups, including the 5-ASA-treated groups, received also oral daily gavage of vehicle (VEH), which consisted of 1X D-PBS that was used either to wash the bacterial pellets, or to resuspend the lyophilized product.

### 4.8. Statistical Analyses

For RT-qPCR data, relative quantification was performed according to the comparative 2^−ΔΔCt^ method [74]. All plots and statistical analyses were performed in GraphPad Prism version 10.3.0 for Windows (GraphPad Software, Boston, MA, USA, www.graphpad.com, accessed on 31 July 2024). Statistical methods are provided with each figure and table. In figures and tables, asterisks indicate statistical significances: *, *p* < 0.05; **, *p* < 0.01; ***, *p* < 0.001; and ****, *p* < 0.0001.

## 5. Conclusions

The role of the gut microbiota in promoting inflammatory bowel disease (IBD) pathology is well recognized. Although there are several treatment options for ulcerative colitis (UC), clinical presentation is very heterogeneous, and the outcome of treatment remains elusive. Indeed, numerous patients exhibit an inadequate response or are not able to tolerate treatment. In addition, most treatments, such as corticosteroids and biologics, result in immune suppression, which has concerning safety risks. Because of the direct role of the gut microbiome in disease, contributing to intestinal epithelial damage and perpetuating inflammation, the potential for microbiome-based therapies is significant, and confirmed by fecal microbiota transplantation (FMT) human trials. However, FMT suffers from a lack of standardization and can potentially transfer unwanted microbiota, including viruses. Therefore, we have developed a six-strain consortium of bacterial commensals (MH002) for the treatment of mild-to-moderate UC. This combination of six strains may be considered a miniature ecosystem optimized to restore butyrate-producing networks, as butyrate is consistently reduced in IBD patients. As a result, MH002, when taken daily, has the potential to promote intestinal epithelium repair and barrier integrity, and to decrease inflammation in vivo. Ultimately, MH002 may prove to be a valid therapeutic option for patients.

## Figures and Tables

**Table 1:** Interleukin (IL) levels (pg/mL) and lactate dehydrogenase (LDH) activity (mU/mL) of medium- and concanavalin A (ConA)-treated Caco-2/PBMCs. ConA-stimulated co-cultures were simultaneously treated with either MH002 cell-free supernatant collected from fed-batch cultures or with 5 mM sodium butyrate (NaB).

		Medium	ConA	ConA + NaB	ConA + MH002
IL-4	Mean ± SEM (*n*)	5.72 ± 1.86 (6)	51.56 ± 10.08 (6)	16.09 ± 1.48 (6)	6.48 ± 4.29 (6)
	*p*-value	0.019	-	0.044	0.040
IL-9	Mean ± SEM (*n*)	1.73 ± 0.63 (6)	296.50 ± 91.89 (6)	11.13 ± 3.13 (6)	10.60 ± 3.67 (6)
	*p*-value	0.056	-	0.059	0.062
IL-17A	Mean ± SEM (*n*)	1.04 ± 0.67 (6)	582.50 ± 88.13 (6)	138.2 ± 26.69 (6)	156.90 ± 37.66 (6)
	*p*-value	0.003	-	0.004	0.010
IL-21	Mean ± SEM (*n*)	2.50 ± 1.54 (6)	79.36 ± 12.60 (6)	13.41 ± 2.20 (6)	6.55 ± 3.92 (6)
	*p*-value	0.005	-	0.008	0.008
IL-22	Mean ± SEM (*n*)	1.21 ± 0.83 (6)	117.80 ± 26.05 (6)	289.4 ± 52.94 (6)	378.40 ± 103.60 (6)
	*p*-value	0.016	-	0.009	0.051
LDH	Mean ± SEM (*n*)	12.31 ± 0.87 (6)	11.88 ± 1.01 (6)	13.15 ± 1.19 (6)	12.36 ± 1.39 (6)
	*p*-value	0.674	-	0.069	0.533

Data are presented as mean ± SE of the mean of PBMCs isolated from six different subjects and tested in three independent experiments. Cytokines and LDH were measured on the basolateral supernatant after 48 h of co-culture. Statistical analyses were performed against ConA by using one-way repeated measures ANOVA with Dunnett’s post hoc test (*p*-values are indicated).

**Table 2:** (A–C) Systemic cytokine levels in the DSS and TNBS colitis studies (all in pg/mL).

(A) Acute DSS mice								
	**Water + VEH**	***n***	**DSS + VEH**	***n***	**DSS + 5-ASA**	***n***	**DSS + MH002**	***n***
G-CSF	0.00 ± 0.00	5	50.03 ± 10.84 **	7	n/a	n/a	29.35 ± 10.90	8
Cxcl1	44.51 ± 11.71	6	160.7 ± 36.66 *	7	n/a	n/a	130.0 ± 31.31	8
IL-6	0.00 ± 0.00	5	27.89 ± 4.92 ***	7	n/a	n/a	19.23 ± 2.67	7
(B) Acute TNBS rats								
	**EtOH + VEH**	***n***	**TNBS + VEH**	***n***	**TNBS + 5-ASA**	***n***	**TNBS + MH002**	***n***
Ifnγ	14.83 ± 1.67	5	43.18 ± 7.47 *	10	15.44 ± 1.43 **	10	31.01 ± 6.87	10
Tnfα	9.89 ± 2.66	5	93.65 ± 35.39	10	17.06 ± 9.89 *	10	16.69 ± 6.71 *	10
IL-2	22.45 ± 5.83	5	160.2 ± 51.45 *	10	16.58 ± 5.36 **	9	37.24 ± 12.22 *	10
(C) Recovery DSS mice								
	**Water + VEH**	***n***	**DSS + VEH**	***n***	**DSS + 5-ASA**	***n***	**DSS + MH002**	***n***
G-CSF	0.00 ± 0.00	5	94.10 ± 11.34 *	5	51.97 ± 20.09	5	56.86 ± 23.95	5
Cxcl1	11.05 ± 3.51	5	149.5 ± 41.03 **	5	37.28 ± 8.74 *	5	50.50 ± 22.61 *	5
IL-6	0.00 ± 0.00	5	184.6 ± 59.97 *	5	92.96 ± 55.11	5	22.57 ± 20.42 *	5

Data are in mean ± SE of the mean. Outliers were removed by using the ROUT method and a false discovery rate = 1%. Asterisks indicate significant difference *, *p* < 0.05; **, *p* < 0.01; ***, *p* < 0.001when compared to the disease + VEH group (one-way ANOVA with Dunnett’s post hoc test). Cxcl1: C-X-C motif chemokine ligand 1; G-CSF: Granulocyte colony stimulating factor; Ifnγ: Interferon gamma; IL: Interleukin; n/a: Not applicable (i.e., group not included in the study); Tnfα: Tumor necrosis factor alpha.

## Data Availability

Data is contained within the article.

## Supplementary Materials

The following supporting information can be downloaded at https://www.mdpi.com/article/10.3390/ijms26136167/s1. References [63,75,76] are cited in the supplementary materials.
