# Supplementary material for: MH002, a Novel Butyrate-Producing Consortium of Six Commensal Bacterial Strains Has Immune-Modulatory and Mucosal-Healing Properties
Source: Int J Mol Sci. 2025 Jun 26;26(13):6167. doi: 10.3390/ijms26136167 (PMC12250263; doi:10.3390/ijms26136167)
Supplement: Supplementary file 1 [file ijms-26-06167-s001.zip › ijms-3597813-supplementary.pdf]

Supplementary Figures and Tables

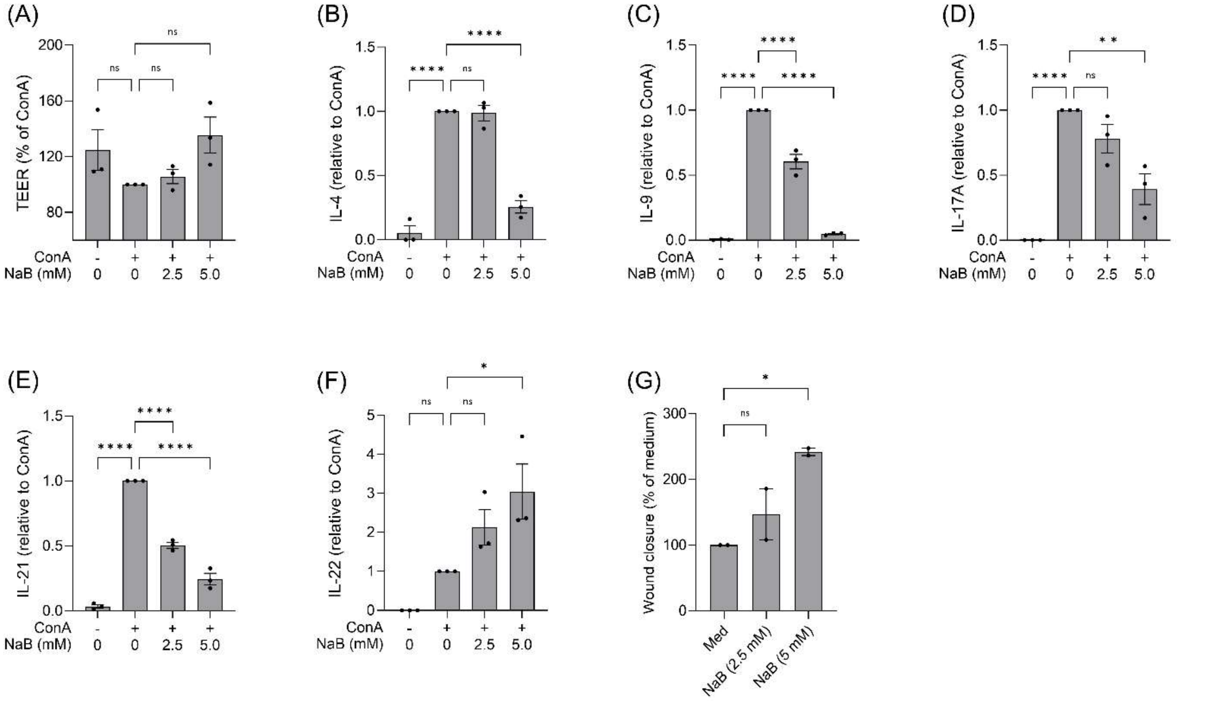

Figure S1: Effect of two doses of sodium butyrate (NaB) on intestinal barrier integrity, cytokine release, and wound repair *in vitro*. **(A)** Transepithelial electrical resistance (TEER) of Caco-2/Peripheral blood mononuclear cells (PBMCs) co-cultures after 48 hours (n=3). **(B-F)** Cytokine levels measured on the basolateral compartment of Caco-2/PBMCs co-cultures (n=3). Results are shown relatively to the average of Concanavalin A (ConA) and present the mean ± SE of the mean of PBMCs isolated from three different subjects and tested in three independent experiments. **(G)** Wound closure in T84 cells measured after 48 hours (n=2). Results are shown relatively to untreated cells (Med, serum-free cell culture medium). Asterisks indicate significant difference (p<0.05) when compared to ConA (one-way ANOVA with Dunnett's post-hoc test). *, p<0.05; **, p<0.01; ***, p<0.001; and ****, p<0.0001.

Table S1: Baseline cytokine levels of medium- and ConA-treated Caco-2/PBMCs tested with the IBD-M-SHIME®-collected samples (all in pg/mL).

		IL-4	IL-9	IL-17A	IL-21	IL-22
	**Mean**	0.65	0.10	0.10	0.15	4.93
**Medium**	**SEM**	0.55	0.00	0.00	0.05	4.83
	**N**	3	3	3	3	3
	**Mean**	62.32	96.26	365.6	31.28	74.49
**ConA**	**SEM**	23.88	31.47	117.8	12.31	27.95
	**N**	3	3	3	3	3

Data are presented as mean ± SE of the mean of PBMCs isolated from three different subjects and tested in three independent experiments. Interleukins (IL) were measured on the basolateral supernatant after 48 hours of co-culture.

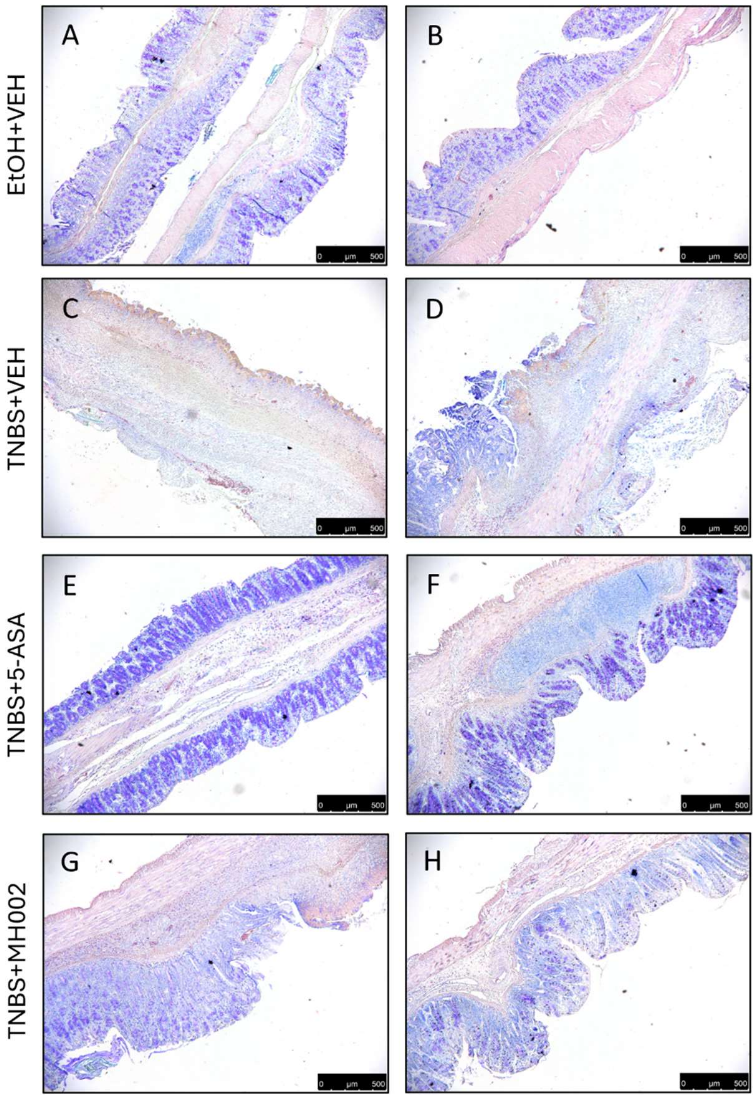

Figure S2: Representative histological features of TNBS-induced acute colitis. In this study colitis was induced by a single intrarectal injection of TNBS; MH002 was administered seven days prior to injection, and until four days after TNBS. The figure shows two representative images per group of Hematoxylin & Eosin-stained colon sections collected at the end of the study from **(A, B)** control healthy mice; **(C, D)** TNBS-treated control mice; **(E, F)** TNBS+5-ASA-treated mice, and **(G, H)** TNBS+MH002-treated mice. TNBS-treated animals show ulceration of the mucosa, edema and loss of the epithelial architecture. This is restored upon 5-ASA and MH002 treatments. Magnification, ×20.

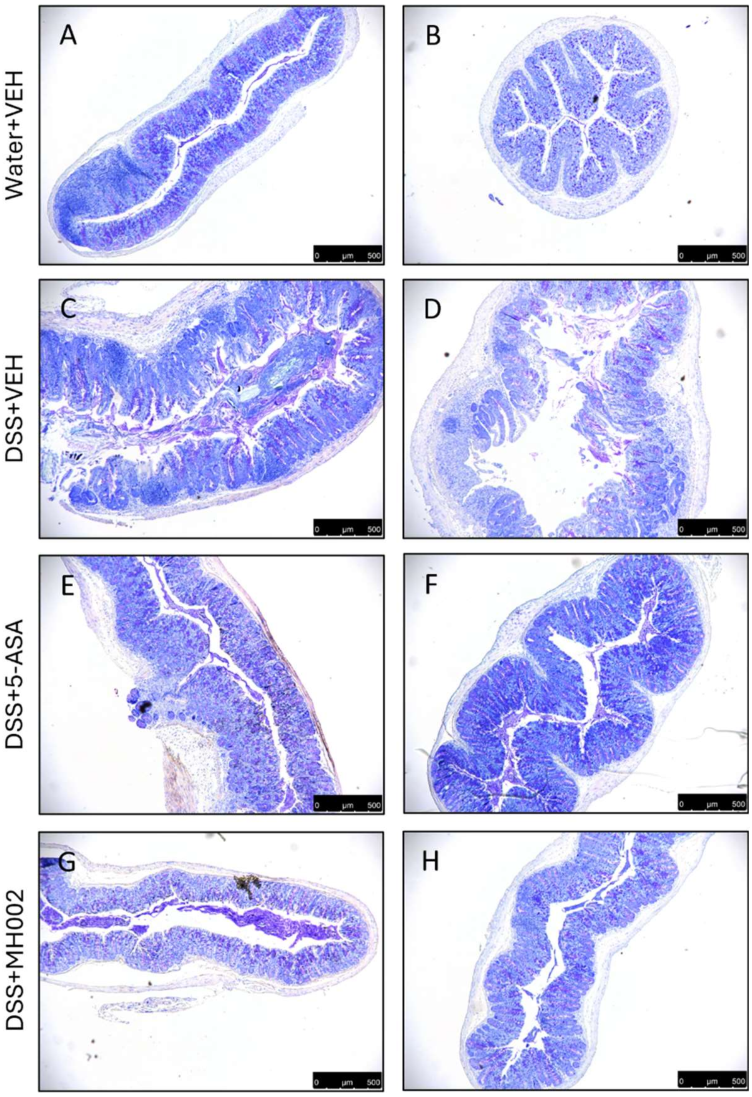

Figure S3: Representative histological features of DSS-induced colitis: recovery DSS study. In this study MH002 was administered for two weeks prior to DSS, during the five days DSS, and until seven days after DSS removal. The figure shows two representative images per group of May-Grünwald-Giemsa-stained colon cross-sections collected at the end of the study from **(A, B)** control healthy mice; **(C, D)** DSS-treated control mice; **(E, F)** DSS+5-ASA-treated mice, and **(G, H)** DSS+MH002-treated mice. DSS-treated animals show loss of epithelial architecture and erosion. This is restored upon 5-ASA and MH002 treatments. Magnification, ×20.

Supplementary Methods

RNA extraction from IBD patient-derived intestinal monolayers and RT-qPCR

Total RNA was extracted from the intestinal monolayers using the RNeasy mini kit (Qiagen, Hilden, Germany), according to the manufacturer's instructions. The concentration and purity of the RNA was determined using nanodrop technology (NanoDrop ND-1000 spectrophotometer, Thermo Fisher Scientific, Waltham, MA, USA). All samples exhibited an OD260/OD280 ratio between 1.8 and 2.2. Then, 250 ng of total RNA was converted to single-stranded complementary (c)DNA by reverse transcription using the High-Capacity cDNA Reverse Transcription kit (Applied Biosystems, Thermo Fisher Scientific, Dilbeek, Belgium), according to the manufacturer's instructions. The cDNA was diluted to a final concentration of 2.5 ng/µL and 5 ng were used in RT-qPCR together with SYBR Green (SensiMix SYBR Low-ROX Kit, Bioline, GC Biotech, Waddinxveen, The Netherlands) and 250 nM of each primer (BioLegio, Nijmegen, The Netherlands). RT-qPCR was performed on a QuantStudio 5 Real Time PCR System (Applied Biosystems, Thermo Fisher Scientific, Waltham, MA, USA) with the following cycling conditions: 95°C for 10 minutes, followed by 40 cycles of 95°C for 15s, 60°C for 15s and 72°C for 20s, and finally, 95°C for 15s, 60°C for 1 min and 95°C for 1s. All reactions were performed in triplicate. A reaction without template was included as negative control. The expression of occludin (*OCLN*), claudin-2 (*CLDN-2*) and mucin-2 (*MUC-2*) were measured and normalized to the housekeeping gene ribosomal protein lateral stalk subunit P0 (*RPLP0*). Primer sequences were as follows (5'-3'):

- *OCLN*: Fw ACAAGCGGTTTTATCCAGAGTC; Rev GTCATCCACAGGCGAAGTTAAT
- *CLDN-2*: Fw CGGGACTTCTACTCACCACTG; Rev GGATGATTCCAGCTATCAGGGA
- *MUC-2*: Fw CGACTACTACAACCCTCCGC; Rev GGGAGGAGTTGGTACACACG
- *RPLP0*: Fw GCAATGTTGCCAGTGTCTG; Rev GCCTTGACCTTTTCAGCAA

Histological assessment of intestinal pathology in the rodent studies

For all studies, distal colon sections of 4 µm were embedded in paraffin and stained with Hematoxylin & Eosin (acute DSS and TNBS) or with May-Grünwald-Giemsa (recovery DSS) and scored in a blinded fashion. In the acute DSS study, the histological score used was a composite score based on Van der Sluis and colleagues [75]. Briefly, sections were scored for epithelial damage (0, none; 1, <20%; 2, 20-50%; 3, 50-70%; 4, 70-100%), mucosa and submucosa cell infiltration, and presence of ulcers. In the TNBS rats, histological samples were evaluated according to the Ameho's criteria [63]. Briefly, this scoring criteria, on a scale from 0-6, grades the degree of inflammation infiltrate, the presence of erosion, ulceration or necrosis, and depth and surface extension of lesions (0, no alterations; 1, mucosal and/or submucosal inflammatory infiltrates with edema, few mucosal erosions, integrity of the *muscularis mucosae*; 2, same as 1, but >50% of the section; 3, large inflammatory infiltrates with ulceration through the colonic wall; 4, same as 3, but >50% of the section; 5, wide ulcerations with cellular necrosis; 6, same as 5, but >50% of the section). Finally, in the recovery DSS study, a composite score (0-14) based on the multiparametric scoring system described by Dieleman and colleagues [76] was used to score the colon sections. This multiparametric scoring system evaluates severity of inflammation (0, none; 1, slight; 2, moderate; 3, severe), extent of epithelial damage (0, none; 1, mucosa; 2, mucosa and submucosa; 3, transmural), crypt damage (0, none; 1, basal 1/3 damaged; 2, basal 2/3 damaged; 3, only surface epithelium intact; 4, entire crypt and epithelium lost) and percentage involvement (1, 1-25%; 2, 25-50%; 3, 50-75%; 4, 75-100%). For all studies, results are expressed as the sum of the individual scores.

RNA isolation and RT-qPCR on distal colon samples

For the acute DSS study, total RNA was extracted from the distal colon using the ISOLATE II RNA Micro Kit (Bioline, GC Biotech, Waddinxveen, The Netherlands), according to the manufacturer's instructions. Concentration and purity of the RNA was determined using the Take3 microvolume plate (Synergy HT Microplate reader, BioTek, Santa Clara, CA, USA). All samples exhibited an OD260/OD280 ratio between 2.0 and 2.2. Then, 1 µg of total RNA was converted to single-stranded cDNA by reverse transcription using the High-Capacity cDNA Reverse Transcription kit (Applied Biosystems, Thermo Fisher Scientific, Dilbeek, Belgium), according to the manufacturer's instructions. The cDNA was diluted to a final concentration of 5 ng/µL and 10 ng were used in RT-qPCR together with SYBR Green (SensiMix SYBR Low-ROX Kit, Bioline, GC Biotech, Waddinxveen, The Netherlands) and 250 nM of each primer (BioLegio, Nijmegen, The Netherlands). RT-qPCR was performed on a QuantStudio 5 Real-Time PCR System (Applied Biosystems, Thermo Fisher Scientific, Waltham, MA, USA) with the following cycling conditions: 95°C for 10 minutes, followed by 40 cycles of 95°C for 15s, 60°C for 15s and 72°C for 20s, and finally, 95°C for 15s, 60°C for 1 min and 95°C for 1s. All reactions were performed in triplicate. A reaction without template was performed in parallel as negative control. All results were normalized to the housekeeping genes actin-beta (*Actb*) and glyceraldehyde 3-phosphate dehydrogenase (*Gapdh*). Primer sequences were as follows (5'-3'):

- Interleukin (*IL*)-*6*: Fw-TAGTCCTTCCTACCCCAATTTCC; Rev-TTGGTCCTTAGCCACTCCTTC
- Occludin (*Ocln*): Fw-CTGGATCTATGTACGGCTCACA; Rev-TCCACGTAGAGACCAGTACCT
- Tight junction protein (*Tjp*)*1*: Fw-GCTTTAGCGAACAGAAGGAGC; Rev-TTCATTTTTCCGAGACTTCACCA
- *Actb*: Fw-CCACTGCCGCATCCTCTTCC; Rev-CTCGTTGCCAATAGTGATGACCTG
- *Gapdh*: Fw-CATGGCCTTCCGTGTTCCTA; Rev- GCGGCACGTCAGATCCA

For the TNBS rat study, total RNA was isolated from the distal colon samples using the RNeasy kit (Macherey Nagel, Hoerdt, France) according to the manufacturer's instructions. RNA quantification was performed using spectrophotometry, and the High-Capacity cDNA Reverse Transcription kit (Applied Biosystems, Thermo Fisher Scientific, Illkirch, France) was used to synthesize single-stranded cDNA. The expression of targeted genes was quantified using SYBR green Master Mix (Applera, Courtaboeuf, France) in a GeneAmp Abiprism 7000 (Applera, Courtaboeuf, France). All reactions were performed in triplicate. In each assay, a reaction without template was included for control. SYBR green dye intensity was analyzed using the Abiprism 7000 SDS software (Applera, Courtaboeuf, France). All results were normalized to the housekeeping gene *Gapdh*. Primer sequences were as follows (5'-3'):

- *IL-6*: Fw-CTTGGAAATGAGAAAAGAGTTGTGC; Rev-TCCAGAAGACCAGAGCAGATTTT
- *Ocln*: Fw-CTTCTTTCCTTAGGCGACCG; Rev-TTGGGTTTGAATTCATCCGGC
- *Tjp1*: Fw-CGGAACTATGACCATCGCCATC; Rev- GCCTGTACCTGTTGTGCACC
- *Gapdh*: Fw-CTGTTCTAGAGACAGCCGCATCT; Rev-ACACCGACCTTCACCATCTTG

Measurement of cytokines in blood samples

For all rodent studies serum levels of cytokines were quantified by Luminex® magnetic beads-based assays (eBioscience, Thermo Fisher Scientific, Dilbeek, Belgium), according to the manufacturer's instructions. These included: murine granulocyte colony-stimulating factor (G-CSF), chemokine (C-X-C motif) ligand 1 (Cxcl1), and interleukin (IL)-6, and rat interferon (Ifn)γ, tumor necrosis factor (Tnf)α, and IL-2. All Luminex® data was recorded in a MAGPIX Instrument (Luminex Corporation, Austin Texas, USA) and analyzed using the ProcartaPlex Analysis App software from Thermo Fisher Scientific (Thermo Fisher Scientific, Waltham, MA, USA). Outliers were removed by using the ROUT method and a False discovery rate = 1%.
